# A Novel FGFR3‐Targeting Antibody‐Drug Conjugate Induces Tumor Cell Apoptosis through the cGAS–STING Pathway in Bladder Cancer

**DOI:** 10.1002/advs.202509933

**Published:** 2025-10-30

**Authors:** Shu Cui, Xiongfei Luo, Guangrui Fan, Jingqi Jiang, Yingru Wang, Enguang Yang, Jinpeng Ma, Ze Zhang, Yuhan Wang, Juan Wang, Dengtuo Wang, Hanzhang Wang, Liang Cheng, Junqiang Tian, Zhilong Dong, Yingqian Liu, Zhiping Wang

**Affiliations:** ^1^ Institute of Urology The Second Hospital of Lanzhou University Gansu Province Clinical Research Center for Urinary System Disease Lanzhou 730030 China; ^2^ School of Pharmacy Lanzhou University Lanzhou 730030 China; ^3^ Robotic Minimally Invasive Surgery Center Sichuan Provincial People's Hospital University of Electronic Science and Technology of China Chengdu 610072 China; ^4^ The Legorreta Cancer Center at Brown University Department of Pathology and Laboratory Medicine The Warren Albert Medical School of Brown University Brown University Health Providence RI USA

**Keywords:** 7‐ethyl‐9‐fluorocamptothecin, antibody‐drug conjugates, bladder cancer, cGAS‐STING, MAD2L1

## Abstract

Antibody‐drug conjugates (ADCs) emerge as a potent cancer therapeutic strategy by enabling precise antigen recognition and efficient intracellular delivery of cytotoxic payloads. In this study, 7‐ethyl‐9‐fluorocamptothecin (A2), a camptothecin derivative, which demonstrates potent tumor‐suppressive effects across cellular models, patient‐derived organoids (PDOs), and cell line‐derived xenograft/patient‐derived xenograft (CDX/PDX) models is identified. Through pull‐down/mass spectrometry analysis, MAD2L1 is identified as the direct target of A2. A2 specifically binds to the Lys73 site of MAD2L1, activating the cGAS‐STING pathway and thereby inducing apoptosis in bladder cancer cells. To address the dose‐limiting toxicity caused by A2's insufficient targeting capability, LZU‐WZLYCS01, a novel FGFR3‐targeting ADC for bladder cancer with A2 as its cytotoxic payload is developed. LZU‐WZLYCS01 exhibits precise FGFR3‐dependent targeting, with significantly reduced antitumor activity in both FGFR3‐knockout cell models and xenograft models. Moreover, in vivo fluorescence imaging demonstrates the potent tumor‐targeting capability of LZU‐WZLYCS01. LZU‐WZLYCS01 demonstrates remarkable bystander effects in an in vitro co‐culture model, along with potent tumor growth inhibition in PDOs and CDX/PDX models while maintaining favorable safety. Notably, LZU‐WZLYCS01 shows superior antitumor efficacy to gemcitabine‐cisplatin (GC) chemotherapy and maintains significant activity in GC‐resistant PDX models. These findings present a promising therapeutic candidate for targeted bladder cancer treatment.

## Graphical abstract

LZU‐WZLYCS01 is a novel FGFR3‐targeting ADC for bladder cancer with 7‐ethyl‐9‐fluorocamptothecin (A2) as its cytotoxic payload. LZU‐WZLYCS01 intracellularly releases A2, which targets MAD2L1 to activate the cGAS‐STING pathway and induce tumor cell apoptosis. LZU‐WZLYCS01 demonstrates potent antitumor efficacy across cellular models, PDOs, and CDX/PDX models. This breakthrough may provide a new strategic option for the precision therapy of bladder cancer.

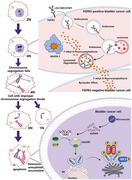

## 1 Introduction

Bladder cancer (BC), one of the most prevalent urological malignancies, demonstrates a particularly poor prognosis in advanced or metastatic stages. The current first‐line therapy primarily relies on platinum‐based combination chemotherapy. However, ≈50% of patients with muscle‐invasive bladder cancer (MIBC) are ineligible for or intrinsically resistant to cisplatin‐based regimens.^[^ ^1^, ^2^ ^]^ Even among initial responders, acquired resistance frequently develops, leading to inevitable disease progression. This therapeutic resistance represents a major cause of treatment failure and cancer‐related mortality in BC. Furthermore, multiple factors, including the lack of effective treatment options after failure of frontline therapy and diverse molecular mechanisms driving resistance, pose formidable clinical challenges in the management of drug‐resistant BC.^[^ ^3^ ^]^ Therefore, innovative therapeutic strategies are urgently needed to improve survival outcomes in patients with BC.

Antibody‐drug conjugates (ADCs), comprising a monoclonal antibody, a linker, and a cytotoxic payload, combine the potent cytotoxic effects of traditional chemotherapy with the target specificity of monoclonal antibodies, thereby providing an innovative strategy to overcome the limitations of conventional chemotherapeutics and targeted agents. Currently, several ADCs, including sacituzumab govitecan (SG), enfortumab vedotin (EV), and disitamab vedotin (RC48), have been approved for the treatment of locally advanced or metastatic BC, demonstrating significant efficacy and safety, particularly in patients who have failed prior immunotherapy.^[^ ^4^, ^5^ ^]^ Despite revolutionizing clinical practice and improving survival outcomes in advanced BC, ADC therapies continue to face significant challenges. First, due to target limitations, their clinical utility remains restricted to patients with target‐positive tumors, limiting overall treatment efficacy. Second, repeated ADC exposure often leads to resistance mechanisms such as antigen downregulation, epitope mutations, and activation of bypass signaling pathways, resulting in a further reduction of available ADC drugs. Therefore, designing and developing a novel ADC targeting highly expressed and tumor‐specific antigens in BC is critical to expand the clinical applications of ADCs and provide more precise and effective treatment strategies for patients.

MAD2L1 is a core component of the spindle assembly checkpoint (SAC), ensuring proper chromosome segregation during mitosis. Recent studies have shown that aberrant expression of MAD2L1 promotes chromosomal instability, tumor heterogeneity, enhanced proliferative capacity, and resistance to chemotherapeutic agents.^[^ ^6^ ^]^ Fibroblast growth factor receptor (FGFR) plays critical roles in various cellular processes including proliferation, differentiation, migration, and apoptosis.^[^ ^7^ ^]^ As a key member, FGFR3 represents one of the most frequently altered targets in BC, with high expression in ≈40% of MIBC and gene mutations in ≈50% of primary BCs.^[^ ^8^, ^9^, ^10^ ^]^ The aberrant activation of FGFR3 is not only a crucial oncogenic driver in BC and other solid tumors, but is also implicated in resistance to both chemotherapy and targeted therapies. Additionally, despite the initial efficacy of FGFR3‐targeted agents like erdafitinib, their clinical benefit is often limited by the emergence of secondary mutations and bypass signaling mechanisms, leading to drug resistance.^[^ ^11^ ^]^ These characteristics establish both FGFR3 and MAD2L1 as compelling therapeutic targets in BC. Consequently, there is an urgent need to develop therapeutic approaches capable of overcoming these molecularly driven challenges.

In our previous study, using camptothecin as the lead structure, we introduced a fluorine atom at the 9‐position and then designed and synthesized a novel camptothecin derivative, 7‐ethyl‐9‐fluorocamptothecin (A2).^[^ ^12^ ^]^ In this study, we found that A2 exhibits potent antitumor activity in BC. Mechanistically, A2 targets MAD2L1 to activate the cGAS‐STING pathway, thereby inducing apoptosis in BC cells. Building on these findings, we developed LZU‐WZLYCS01, a novel FGFR3‐targeting ADC for BC, with A2 as its highly potent cytotoxic payload. LZU‐WZLYCS01 is designed to achieve precise drug delivery through FGFR3 targeting while simultaneously leveraging A2‐mediated MAD2L1 inhibition to eradicate tumor cells, providing a promising therapeutic strategy to address these molecularly driven challenges.

## 2 Results

### 2.1 In Vitro and In Vivo Antitumor Activity of A2

**Figure 1:**
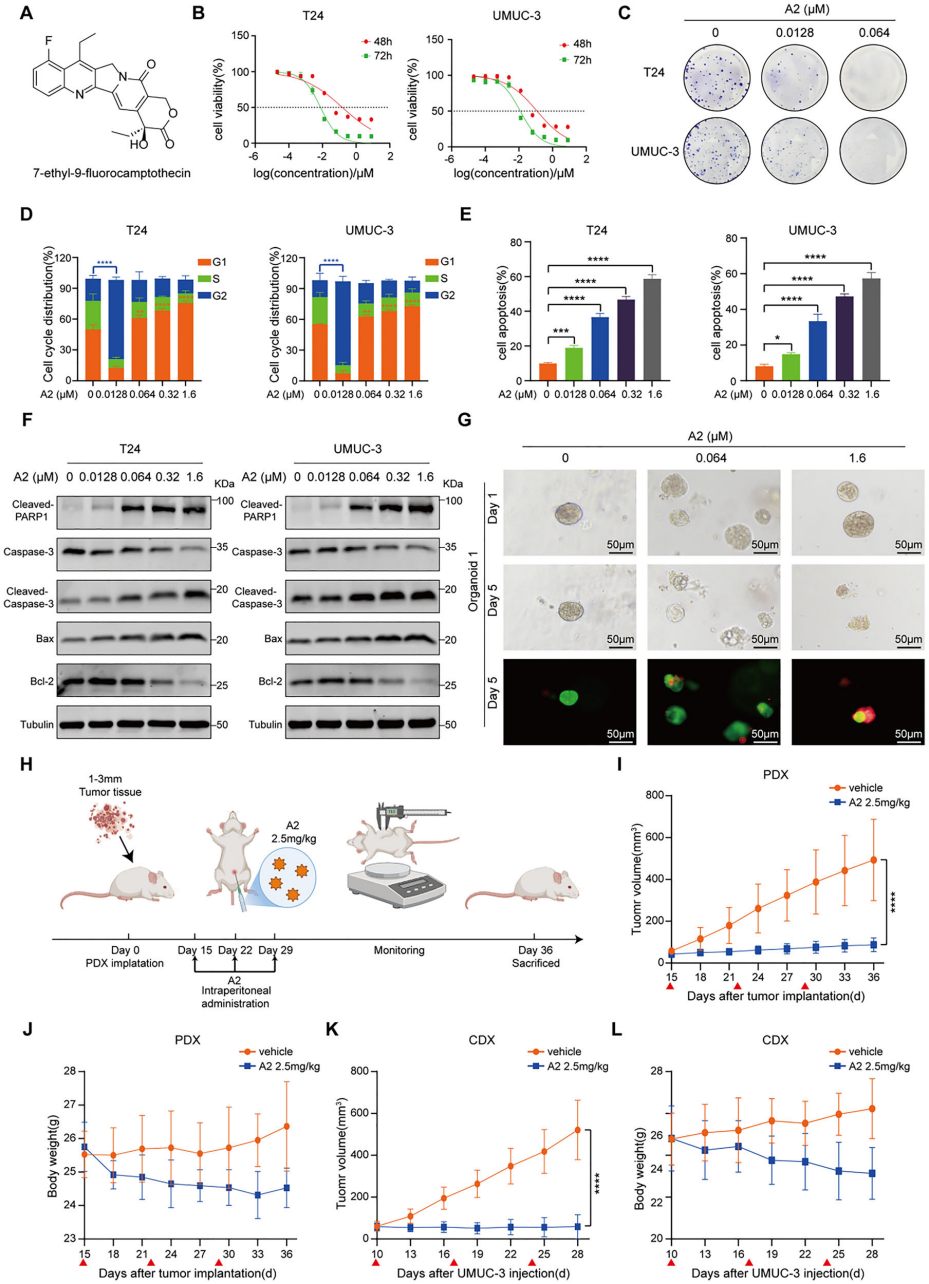
In vitro and in vivo antitumor activity of A2. A) Schematic structure of A2. B) Cell viability of T24 and UMUC‐3 cells treated with different concentrations of A2 for 48 or 72 h. C) Colony formation capacity of T24 and UMUC‐3 cells treated with different concentrations of A2 for 48 h. D) Cell cycle distribution in T24 and UMUC‐3 cells treated with different concentrations of A2 for 24 h (*n* = 3). E) Apoptotic effects in T24 and UMUC‐3 cells treated with different concentrations of A2 for 48 h (*n* = 3). F) Western blot analysis of apoptosis‐related proteins in T24 and UMUC‐3 cells treated with different concentrations of A2 for 48 h. G) Representative bright‐field and AM/PI‐stained images showing A2‐induced cytotoxicity in PDOs. Green: viable cells, red: dead cells, scale bar = 50 µm. H) Experimental flowchart of A2 treatment in PDX tumors. I) Growth curves of PDX tumors (*n* = 5). J) Changes in mouse body weight during A2 treatment. K) Growth curves of UMUC‐3 xenograft tumors (*n* = 5). L) Changes in mouse body weight during A2 treatment. A2 was administered intraperitoneally once weekly for 3 doses (indicated by ▲). Data are presented as the mean ± SD. *P < 0.05, **P < 0.01, ***P < 0.001, ****P < 0.0001. Statistical significance was determined by one‐way ANOVA followed by Tukey's multiple comparison test (D,E) and two‐way ANOVA followed by Tukey's multiple comparison test (I,K).

In our previous study, we designed and synthesized a novel camptothecin derivative, A2 (**Figure** **1**A), which demonstrated potent antitumor activity across multiple malignancies. To evaluate its efficacy in BC, we treated T24 and UMUC‐3 cells with different concentrations of A2. CCK‐8 assays revealed that A2 significantly reduced cell viability in a time‐ and dose‐dependent manner (Figure 1B). The IC_50_ values for T24 and UMUC‐3 were 0.152 and 0.120 µm at 48 h, and 0.008 and 0.012 µm at 72 h, respectively. Notably, the cytotoxicity of A2 was considerably greater than that of topotecan and camptothecin, which exhibited 72 h IC_50_ values of 0.242 and 0.054 µm in T24 cells, respectively (Figure S1A, Supporting Information). Colony formation assays showed that A2 dose‐dependently inhibited clonogenicity of T24 and UMUC‐3 cells (Figure 1C). Flow cytometry cell cycle analysis indicated that low concentrations of A2 induced G2/M arrest, whereas high concentrations led to G1 arrest in T24 and UMUC‐3 cells (Figure 1D; Figure S1B, Supporting Information). Apoptosis assays confirmed that A2 induced apoptosis in T24 and UMUC‐3 cells in a dose‐dependent manner, with apoptosis rates of 58.7% and 57.4% at 1.6 µm A2, respectively (Figure 1E; Figure S1C, Supporting Information). In contrast, Topotecan at the same concentration (1.6 µm) induced markedly lower apoptosis rates of 31.6% and 28.4% in T24 and UMUC‐3 cells, respectively (Figure S1D, Supporting Information). Western blot demonstrated that A2 treatment for 48 h upregulated the expression of Cleaved‐PARP1, Cleaved‐Caspase‐3, and BAX, while downregulating Caspase‐3 and Bcl‐2 in T24 and UMUC‐3 cells (Figure 1F). To directly rule out the involvement of necroptosis, we examined the key executioner protein p‐MLKL by Western blot in T24 and UMUC‐3 cells. The results demonstrated that A2 treatment did not upregulate p‐MLKL levels at any concentration. In contrast, TSZ (a combination of TNF‐α, a Smac mimetic, and Z‐VAD‐FMK), a known necroptosis inducer,^[^ ^13^, ^14^ ^]^ significantly upregulated p‐MLKL levels in T24 and UMUC‐3 cells (Figure S2, Supporting Information). To evaluate the cytotoxic effects of A2 on patient‐derived organoids (PDOs), we treated them with increasing concentrations for 5 days. Inverted phase‐contrast microscopy and live/dead cell staining revealed marked disintegration, fragmentation, and death in all three PDO models, with enhanced cytotoxicity at higher A2 concentrations (Figure 1G; Figure S3, Supporting Information). We further evaluated the antitumor activity of A2 in gemcitabine‐cisplatin (GC)‐resistant patient‐derived xenograft (PDX) models of BC and UMUC‐3 xenograft models. Mice were intraperitoneally administered 2.5 mg kg^−1^ A2 (once weekly for 3 doses), and tumor volumes were measured every three days after treatment initiation (Figure 1H). The results demonstrated that A2 significantly inhibited tumor growth not only in GC‐resistant PDX models (Figure 1I; Figure S4A, Supporting Information) but also in UMUC‐3 xenograft models (Figure 1K; Figure S4B, Supporting Information). Immunohistochemistry (IHC) analysis revealed that A2 treatment decreased the expression of MAD2L1 and Ki‐67 in tumor tissues (Figure S5A, Supporting Information). To assess the safety of A2 treatment, we monitored changes in mouse body weight during the treatment period and performed Hematoxylin and Eosin (H&E) staining on collected organ tissues after treatment completion. As shown in Figure 1J,L, mouse body weight showed a declining trend compared with pre‐treatment levels, and H&E staining indicated mild multifocal necrosis with inflammatory cell infiltration in the liver following A2 treatment (Figure S5B, Supporting Information). These findings indicate that A2 exhibits potent antitumor activity but may induce certain toxic side effects due to its non‐selective action on normal tissues.

### 2.2 A2 Induces Apoptosis in BC Cells by Targeting MAD2L1

**Figure 2:**
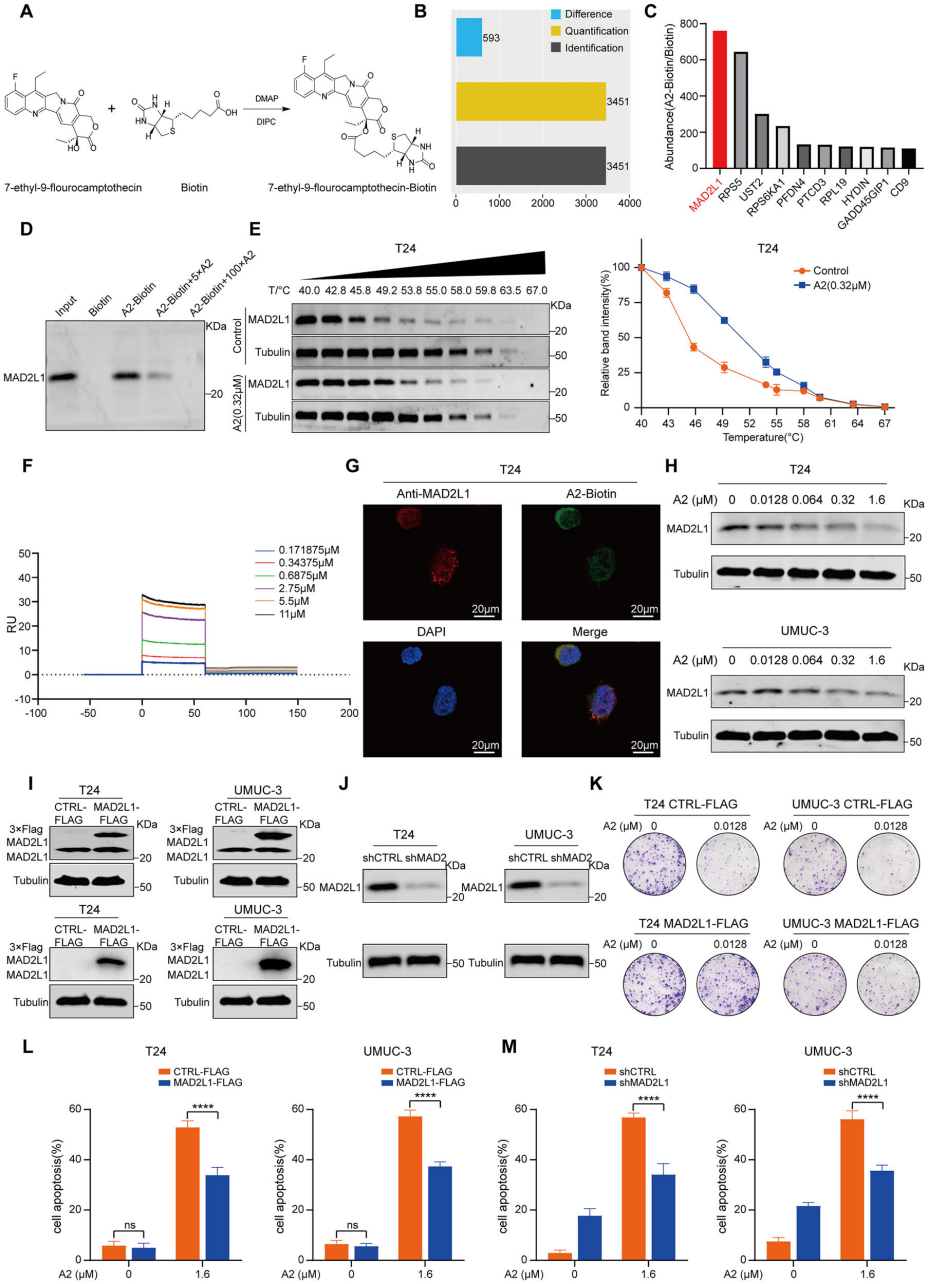
A2 induces apoptosis in BC cells by targeting MAD2L1. A) Reaction scheme for biotinylation at the 20th hydroxyl group of A2. B) Overview of differentially expressed proteins identified by MS. C) Top 10 proteins ranked by the A2‐biotin/biotin abundance ratio. D) Western blot analysis of MAD2L1 expression in pull‐down products. E) Western blot detection of soluble MAD2L1 in T24 cells treated with A2 at different temperatures (*n* = 3). F) SPR analysis of A2‐MAD2L1 binding kinetics and KD value. G) Representative immunofluorescence images showing co‐localization of A2‐biotin and MAD2L1 in T24 cells after 3 h of A2‐biotin treatment. Red: Anti‐MAD2L1, green: A2‐biotin, blue: DAPI, scale bar = 20 µm. H) Western blot analysis of MAD2L1 expression in T24 and UMUC‐3 cells treated with different concentrations of A2 for 24 h. I) Western blot validation of MAD2L1 OE in T24 and UMUC‐3 cells. J) Western blot validation of MAD2L1 KD in T24 and UMUC‐3 cells. K) Colony formation capacity of MAD2L1‐OE and control cells treated with different concentrations of A2 for 48 h. L) Apoptotic effects in MAD2L1‐OE and control cells treated with different concentrations of A2 for 48 h. (*n* = 3). M) Apoptotic effects in MAD2L1‐KD and control cells treated with different concentrations of A2 for 48 h (*n* = 3). Data are presented as mean ± SD. ****P < 0.0001. Statistical significance was determined by two‐way ANOVA followed by Tukey's multiple comparison test (L,M).

To identify the direct target of A2, we biotinylated its 20th hydroxyl group (**Figure** **2**A) and performed pull‐down assays coupled with mass spectrometry (MS) to identify potential binding proteins. A total of 593 differentially expressed proteins were identified (Figure 2B). Among them, MAD2L1 exhibited the highest abundance ratio between A2‐biotin and biotin groups (Figure 2C lists the top 10 proteins with the highest abundance ratios). To evaluate the association between MAD2L1 expression and clinicopathological features in BC patients, we analyzed transcriptomic data from the Cancer Genome Atlas (TCGA) database. Notably, MAD2L1 expression was significantly upregulated in BC (Figure S6A, Supporting Information). Further analysis of Gene Expression Omnibus (GEO) data revealed that high MAD2L1 expression was significantly associated with poor overall survival (OS) in BC patients (Figure S6B, Supporting Information). Subsequent pull‐down/Western blot analysis demonstrated that A2‐biotin directly binds to MAD2L1, and this binding was competitively inhibited by an excess of unlabeled A2 monomer (Figure 2D). Cellular thermal shift assay (CETSA) showed that A2 treatment (0.32 µm) increased the thermal stability of MAD2L1 in a temperature‐dependent manner compared with controls (Figure 2E). Surface plasmon resonance (SPR) analysis further supported the direct interaction between A2 and MAD2L1, with a dissociation constant (KD) of 5.02E‐06 (Figure 2F). Immunofluorescence staining revealed co‐localization of A2‐biotin and MAD2L1 in T24 cells (Figure 2G). To determine whether A2 affects MAD2L1 protein expression, T24 and UMUC‐3 cells were treated with varying concentrations of A2 for 24 h. Western blot results showed that A2 treatment significantly reduced MAD2L1 protein levels in T24 and UMUC‐3 cells (Figure 2H). To further investigate the effects of A2 on MAD2L1 function, we generated stable MAD2L1‐overexpressing (MAD2L1‐OE) and MAD2L1‐knockdown (MAD2L1‐KD) clones in T24 and UMUC‐3 cells (Figure 2I,J). Colony formation assays demonstrated that, at the same drug concentration, MAD2L1‐OE cells formed significantly more colonies than control cells (Figure 2K). Apoptosis assays revealed that MAD2L1‐OE cells exhibited reduced apoptosis compared with control cells, with apoptosis rates decreasing by 19% and 19.9% in T24 and UMUC‐3 cells, respectively, at 1.6 µm A2 (Figure 2L; Figure S7A, Supporting Information). Similarly, MAD2L1‐KD cells also showed a significant reduction in apoptosis relative to controls, with decreases of 22.7% and 20.4% in T24 and UMUC‐3 cells, respectively, at the same concentration (Figure 2M; Figure S7B, Supporting Information). These results collectively indicate that A2 induces apoptosis in BC cells by targeting MAD2L1.

### 2.3 A2 Specifically Binds to the Lys73 Site of MAD2L1

**Figure 3:**
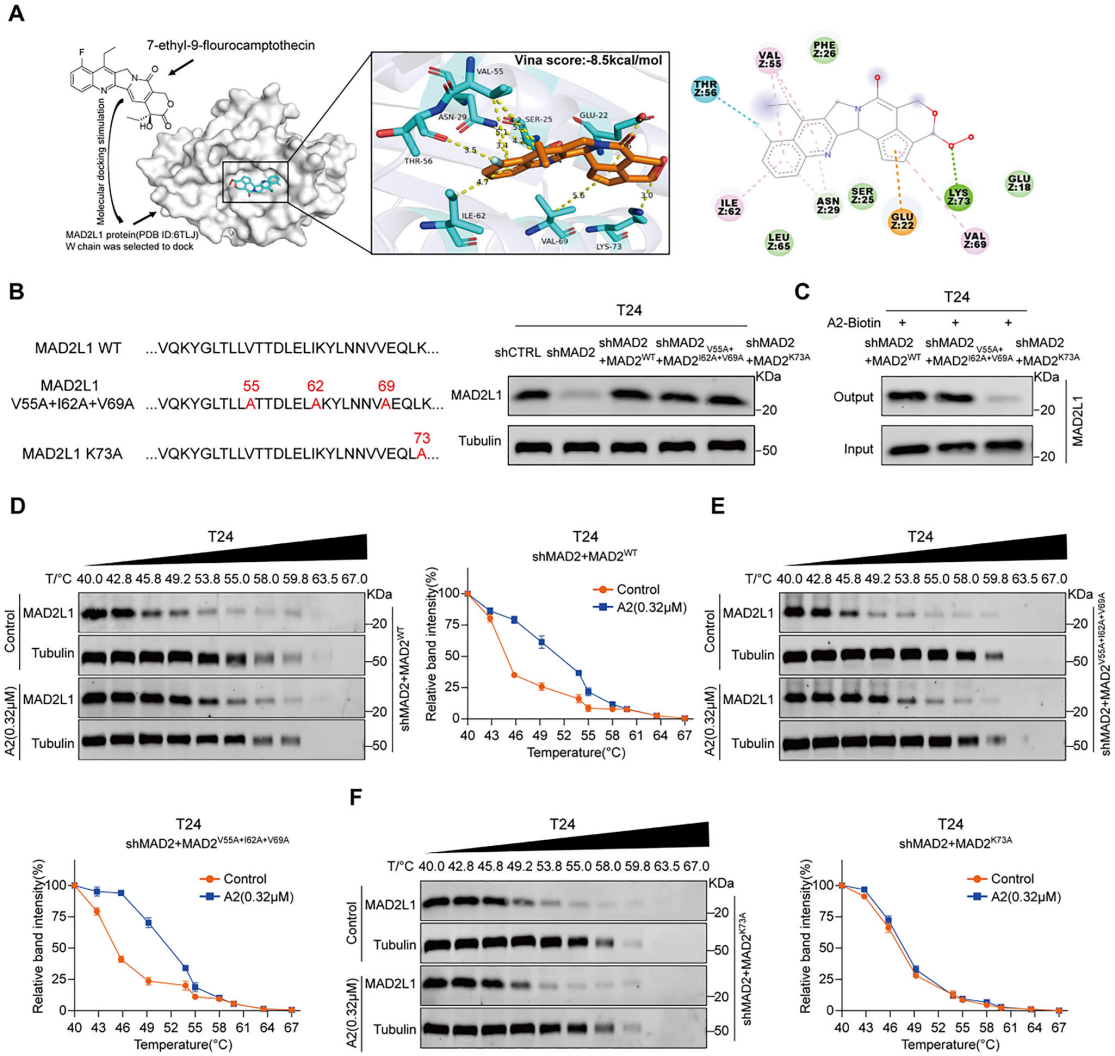
A2 specifically binds to the Lys73 site of MAD2L1. A) Molecular docking simulation of the interaction between A2 and MAD2L1. B) Western blot validation of MAD2L1 expression levels in control, MAD2L1‐KD, and mutant T24 cells. C) Western blot analysis of MAD2L1 expression in pull‐down products from each group. D–F CETSA evaluating the binding of A2 to MAD2L1 in MAD2L1‐KD T24 cells reconstituted with MAD2L1 WT (D), MAD2L1V55A+I62A+V69A (E), and MAD2L1K73A (F) (*n* = 3). Data are presented as mean ± SD.

To further identify the binding site of A2 on MAD2L1, we performed molecular docking simulations to predict the interaction between A2 and MAD2L1. The results indicated that A2 fully occupies the active site of MAD2L1, with a binding energy of −8.5 kcal mol^−1^. A2 is likely to bind to MAD2L1 through the formation of a hydrogen bond with Lys73 and hydrophobic interactions with Val55, Ile62, and Val69 (**Figure** **3**A). We reconstituted wild‐type MAD2L1 (MAD2L1WT) and a series of mutants (MAD2L1V55A+I62A+V69A, MAD2L1K73A) in MAD2L1‐KD T24 cells, and validated MAD2L1 expression by Western blot (Figure 3B). Subsequent pull‐down/Western blot analysis confirmed that only the MAD2L1K73A mutation significantly attenuated the binding between A2 and MAD2L1 (Figure 3C). Furthermore, CETSA results demonstrated that in T24 MAD2L1WT cells, A2 treatment (0.32 µm) enhanced the thermal stability of MAD2L1 in a temperature‐dependent manner compared with the control group (Figure 3D). In T24 MAD2L1V55A+I62A+V69A cells, A2 treatment still enhanced the thermal stability of MAD2L1 (Figure 3E). However, in T24 MAD2L1K73A cells, the enhancement of MAD2L1 thermal stability by A2 was markedly diminished or abolished (Figure 3F), indicating that the Lys73 residue is essential for the binding between A2 and MAD2L1.

### 2.4 A2 Targets MAD2L1 to Activate the cGAS‐STING Pathway

**Figure 4:**
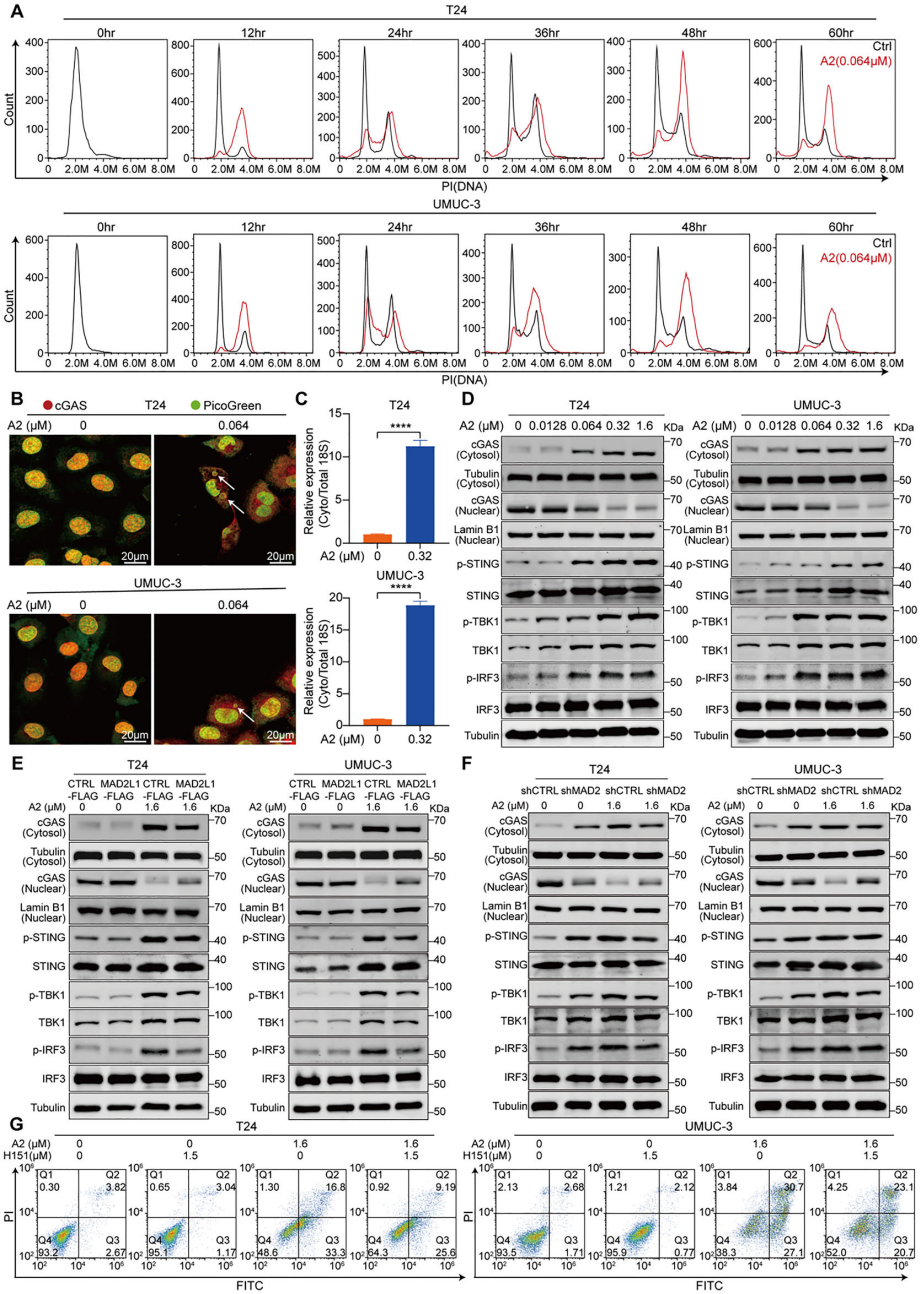
A2 targets MAD2L1 to activate the cGAS‐STING pathway. A) Effect of A2 on aneuploidy formation in T24 and UMUC‐3 cells. B) Representative images showing cGAS subcellular distribution and micronuclei formation (indicated by arrows) in T24 and UMUC‐3 cells treated with 0.064 µm A2 for 48 h. Red: cGAS, green: PicoGreen (DNA stain), Scale bar = 20 µm. C) Quantification of cytosolic DNA in T24 and UMUC‐3 cells treated with 0.32 µm A2 for 48 h (*n* = 3). D) Western blot analysis of cGAS‐STING pathway protein expression in T24 and UMUC‐3 cells treated with different concentrations of A2 for 48 h. E) Western blot analysis of cGAS‐STING pathway protein expression in MAD2L1‐OE and control cells treated with different concentrations of A2 for 48 h. F) Western blot analysis of cGAS‐STING pathway protein expression in MAD2L1‐KD and control cells treated with different concentrations of A2 for 48 h. G) Apoptotic effects in T24 and UMUC‐3 cells treated with 1.6 µm A2 after STING inhibitor H151‐mediated blockade of the cGAS‐STING pathway. Data are presented as mean ± SD. ****P < 0.0001. Statistical significance was determined by a two‐tailed Student's *t‐*test (C).

A2 targets MAD2L1, leading to SAC dysfunction, which causes chromosomal missegregation, aneuploidy, and subsequent micronuclei formation.^[^ ^15^, ^16^ ^]^ To elucidate the molecular mechanism by which A2 induces BC cell apoptosis through targeting MAD2L1, we profiled the chromosome content in T24 and UMUC‐3 cells treated with A2. The results showed that A2 increased the proportion of aneuploid cells in both T24 and UMUC‐3 cells (**Figure** **4**A). PicoGreen staining revealed the formation of micronuclei (indicated by arrows) in T24 and UMUC‐3 cells after A2 treatment (Figure 4B). Micronuclei membranes are prone to irreversible rupture, exposing micronuclear genomic DNA in the cytoplasm and activating the cellular DNA‐sensing pathway.^[^ ^17^ ^]^ Our study found that A2 treatment significantly increased cytoplasmic DNA in T24 and UMUC‐3 cells (Figure 4C). It has been widely accepted that cGAS is unresponsive to self‐DNA due to its cytosolic localization.^[^ ^18^ ^]^ However, recent studies have revealed that cGAS is predominantly localized in the nucleus, where tight chromatin tethering keeps it inactive.^[^ ^19^, ^20^ ^]^ When aberrant DNA appears in the cytoplasm, it triggers the translocation of nuclear cGAS to the cytoplasm, thereby activating the cGAS‐STING pathway.^[^ ^21^ ^]^ In our study, we observed that cGAS is primarily localized in the nucleus in both T24 and UMUC‐3 cells. However, after 48 h of A2 treatment, cGAS levels significantly decreased in the nucleus and increased in the cytoplasm (Figure 4B). This result was further confirmed by Western blot analysis of nuclear and cytoplasmic fractions for cGAS (Figure 4D). Additionally, we examined other key proteins in the cGAS‐STING pathway and found that A2 upregulated the expression of p‐TBK1, p‐IRF3, and p‐STING (Figure 4D). We then treated MAD2L1‐OE and control T24 and UMUC‐3 cells with A2 for 48 h and observed the changes in these proteins. Western blot results revealed that, at the same drug concentration, the protein levels of cGAS in the cytoplasm and of p‐TBK1, p‐IRF3, and p‐STING were lower in MAD2L1‐OE cells compared with the control cells (Figure 4E). Consistently, similar results were observed in MAD2L1‐KD cells (Figure 4F). These findings indicate that A2 activates the cGAS‐STING pathway to inhibit tumor growth by targeting MAD2L1. Activation of the cGAS–STING pathway triggers downstream signaling cascades that lead to apoptosis.^[^ ^22^, ^23^ ^]^ To confirm the relationship between the cGAS‐STING pathway and apoptosis, we blocked the pathway using a STING inhibitor and assessed A2‐induced apoptosis by flow cytometry. The results showed that A2‐induced apoptosis in T24 and UMUC‐3 cells was significantly reduced after STING inhibition (Figure 4G).

### 2.5 Design and Synthesis of LZU‐WZLYCS01, a Novel FGFR3‐Targeting ADC with A2 as the Cytotoxic Payload

**Figure 5:**
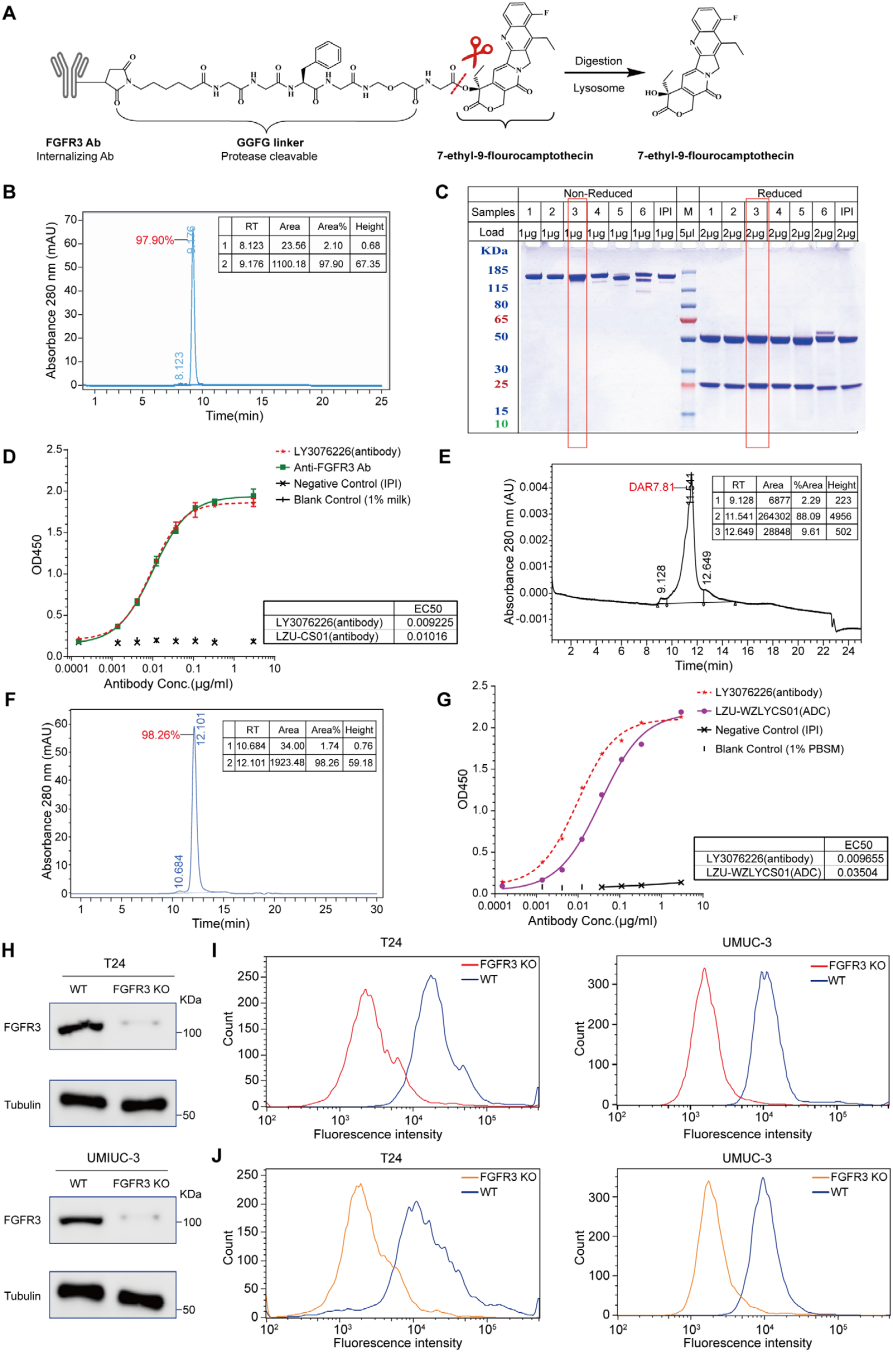
Design and synthesis of LZU‐WZLYCS01, a novel FGFR3‐targeting ADC with A2 as the cytotoxic payload. A) Schematic structure of LZU‐WZLYCS01. B) SEC‐HPLC analysis of FGFR3 antibody purity. C) SDS‐PAGE analysis of FGFR3 antibody under reducing and non‐reducing conditions. D) ELISA evaluation of the binding activity of the FGFR3 antibody to its antigen (*n* = 3). E) DAR determination of LZU‐WZLYCS01 by HIC. F) SEC‐HPLC analysis of LZU‐WZLYCS01 purity. G) ELISA evaluation of the binding activity of LZU‐WZLYCS01 to FGFR3 antigen. H) Western blot validation of FGFR3‐KO in T24 and UMUC‐3 cells. I) Flow cytometry analysis of FGFR3 expression in WT and FGFR3‐KO T24 and UMUC‐3 cells. J) Flow cytometry analysis of the binding activity of LZU‐WZLYCS01 to FGFR3 antigen on WT and FGFR3‐KO T24 and UMUC‐3 cells. Data are presented as the mean ± SD.

Given the potent antitumor activity of A2 but its off‐target toxicity in normal tissues such as the gastrointestinal tract and liver due to a lack of targeting ability, we designed and developed LZU‐WZLYCS01, a novel ADC targeting FGFR3, a BC‐specific antigen, with A2 as the cytotoxic payload (**Figure** **5**A). This design aimed to retain the potent antitumor activity of A2 while minimizing off‐tumor toxicity.

Based on a systematic evaluation of existing FGFR3 monoclonal antibodies, we selected the recombinant humanized IgG1 FGFR3 antibody LY3076226 (Eli Lilly).^[^ ^24^ ^]^ To ensure antibody quality, we assessed its purity using SEC‐HPLC, which showed a purity of 97.9% (Figure 5B). SDS‐PAGE analysis demonstrated that our prepared FGFR3 antibody (boxed in Figure 5C) was consistent with the reference antibody Ipilimumab (IPI) in molecular weight and light/heavy chain ratios under both reducing and non‐reducing conditions. Enzyme‐linked immunosorbent assay (ELISA) further evaluated the antibody's binding ability, revealing that its affinity for the FGFR3 antigen was comparable to that of LY3076226 (Figure 5D). Limulus amebocyte lysate (LAL) testing confirmed endotoxin levels of <1 EU mg^−1^ in the FGFR3 antibody. After confirming that the antibody met quality standards, we conjugated it with the linker‐payload (LP) via thiol‐based coupling to produce LZU‐WZLYCS01. Hydrophobic interaction chromatography (HIC) revealed homogeneous drug distribution, and the drug‐to‐antibody ratio (DAR) of LZU‐WZLYCS01 was ≈7.81 (Figure 5E). SEC‐HPLC analysis demonstrated a purity of 98.26% for LZU‐WZLYCS01 (Figure 5F). ELISA results confirmed that LZU‐WZLYCS01 exhibited FGFR3 antigen binding affinity comparable to that of LY3076226 (Figure 5G), indicating that conjugation of the FGFR3 antibody with A2 did not compromise its biological activity. LAL testing confirmed endotoxin levels of <1 EU mg^−1^. To investigate whether LZU‐WZLYCS01's cytotoxicity in BC cells depends on FGFR3 expression, we generated stable FGFR3‐knockout (FGFR3‐KO) clones in T24 and UMUC‐3 cells (Figure 5H). Flow cytometry results were consistent with Western blot data, showing nearly complete loss of FGFR3 protein in KO cells (Figure 5I). Flow cytometry further demonstrated that LZU‐WZLYCS01 bound efficiently to wild‐type (WT) T24 and UMUC‐3 cells but showed minimal binding to FGFR3‐KO cells (Figure 5J).

### 2.6 Cytotoxicity of LZU‐WZLYCS01 against BC Cells and PDOs, and Validation of Bystander Effect

**Figure 6:**
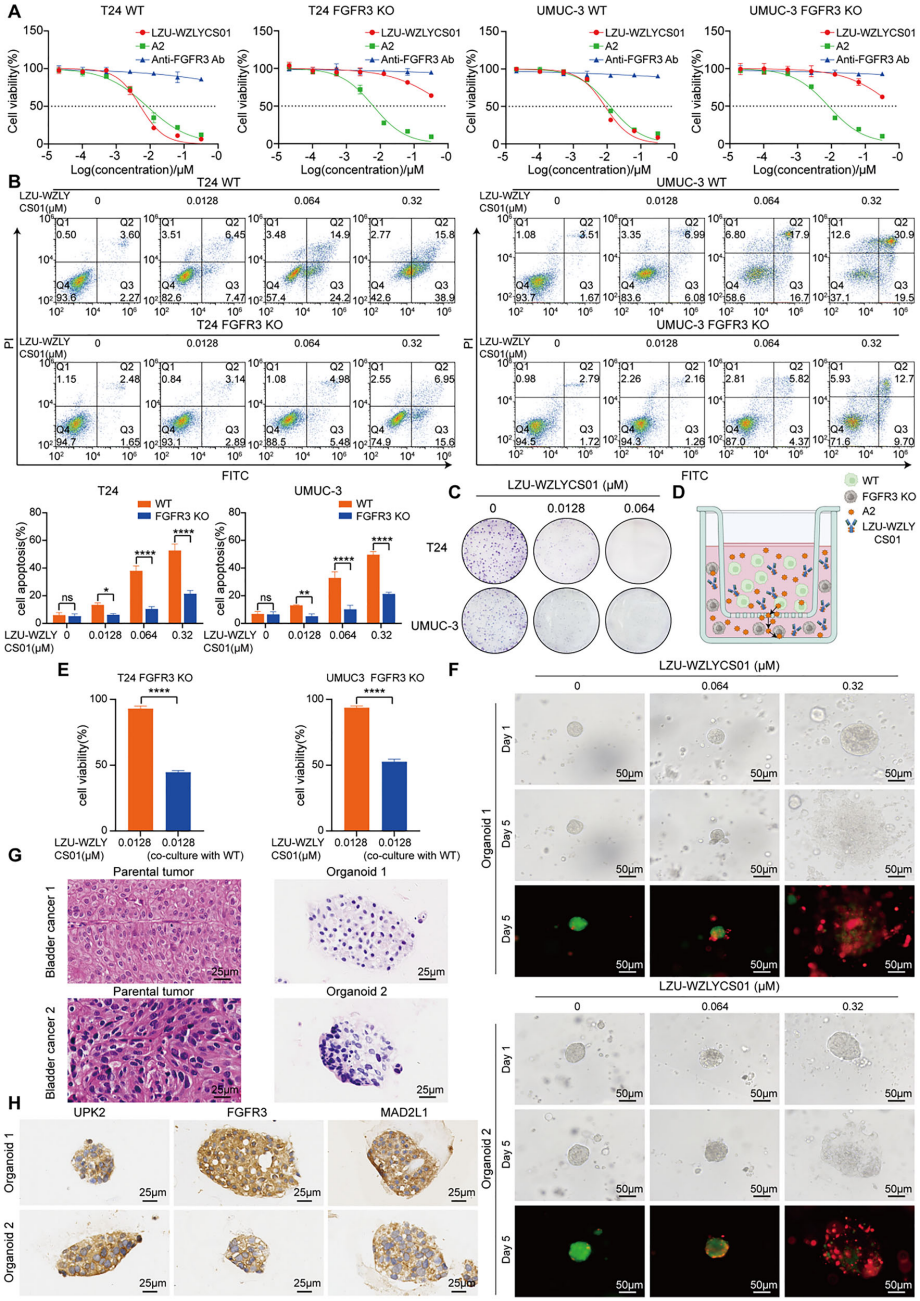
Cytotoxicity of LZU‐WZLYCS01 against BC cells and PDOs, and validation of the bystander effect. A) Cell viability of WT and FGFR3‐KO T24 and UMUC‐3 cells treated with different concentrations of LZU‐WZLYCS01, A2, or FGFR3 antibody for 72 h. B) Apoptotic effects in WT and FGFR3‐KO T24 and UMUC‐3 cells treated with different concentrations of LZU‐WZLYCS01 for 48 h (*n* = 3). C) Colony formation capacity of T24 and UMUC‐3 cells treated with LZU‐WZLYCS01 for 48 h. D) Schematic diagram of the co‐culture model for assessing the bystander effects. E) Effect of LZU‐WZLYCS01 on the viability of FGFR3‐KO T24 and UMUC‐3 cells in the co‐culture system (*n* = 3). F) Representative bright‐field and AM/PI‐stained images showing LZU‐WZLYCS01‐induced cytotoxicity in PDOs. Green: viable cells, red: dead cells, scale bar = 50 µm. G) Representative H&E staining of PDOs and matched parental tumor tissues. Scale bar = 25 µm. H) Representative IHC staining of UPK2, FGFR3, and MAD2L1 in PDOs. Scale bar = 25 µm. Data are presented as mean ± SD. *P < 0.05, **P < 0.01, ****P < 0.0001. Statistical significance was determined by two‐way ANOVA followed by Tukey's multiple comparison test (B) and a two‐tailed Student's *t‐*test (E).

We first treated WT and FGFR3‐KO T24 and UMUC‐3 cells with varying concentrations of LZU‐WZLYCS01, A2, and anti‐FGFR3 antibody. CCK‐8 results revealed that both WT and FGFR3‐KO T24 and UMUC‐3 cells were sensitive to A2. The 72 h IC_50_ values were 0.008 and 0.006 µm for T24 WT and KO cells, and 0.012 and 0.008 µm for UMUC‐3 WT and KO cells, respectively. LZU‐WZLYCS01 exhibited significant growth inhibition in WT T24 and UMUC‐3 cells, with 72h IC_50_ values of 0.005 and 0.009 µm, respectively. However, its inhibitory activity was markedly reduced in FGFR3‐KO T24 and UMUC‐3 cells. In contrast, the anti‐FGFR3 antibody showed no growth inhibition in either WT or FGFR3‐KO cells (**Figure** **6**A). Collectively, these findings suggest that LZU‐WZLYCS01 exerts cytotoxic activity that is both FGFR3‐dependent and payload‐dependent. Flow cytometric apoptosis assays demonstrated that LZU‐WZLYCS01 induced apoptosis in WT T24 and UMUC‐3 cells in a dose‐dependent manner. At 0.32 µm, apoptosis rates were 52.7% and 49.8% for WT T24 and UMUC‐3 cells, respectively. In contrast, apoptosis was significantly reduced in FGFR3‐KO cells at the same concentration. The apoptosis rates were 21.6% and 21.4% for FGFR3‐KO T24 and UMUC‐3 cells at 0.32 µm, respectively (Figure 6B). Colony formation assays confirmed that LZU‐WZLYCS01 significantly inhibited clonogenicity in T24 and UMUC‐3 cells in a dose‐dependent manner (Figure 6C). The bystander effect, where the cytotoxic payload released from antigen‐positive tumor cells diffuses into the tumor microenvironment and kills adjacent antigen‐negative cells, is a critical feature of ADCs.^[^ ^25^ ^]^ To assess whether LZU‐WZLYCS01 induces this effect, we established an in vitro co‐culture model (Figure 6D). Results showed that 0.0128 µm LZU‐WZLYCS01 had no significant cytotoxicity on FGFR3‐KO T24 and UMUC‐3 cells alone. However, in the co‐culture system, the same concentration of LZU‐WZLYCS01 significantly reduced the viability of FGFR3‐KO cells, indicating a clear bystander effect (Figure 6E). To evaluate the efficacy of LZU‐WZLYCS01 in PDOs, we treated them with varying concentrations of LZU‐WZLYCS01 for 5 days. Inverted phase‐contrast microscopy and live/dead cell staining revealed marked disintegration, fragmentation, and death in all three PDO models, with enhanced cytotoxicity at higher LZU‐WZLYCS01 concentrations (Figure 6F; Figure S8, Supporting Information). H&E staining of the PDOs and their corresponding parental tumors demonstrated strong concordance in histopathological features (Figure 6G). IHC confirmed positive expression of UPK2 (a definitive marker of urothelial differentiation), FGFR3, and MAD2L1 in the PDO models (Figure 6H), supporting their suitability for evaluating LZU‐WZLYCS01's therapeutic efficacy.

### 2.7 Mechanism of Action and Tumor‐Targeting Capability of LZU‐WZLYCS01

**Figure 7:**
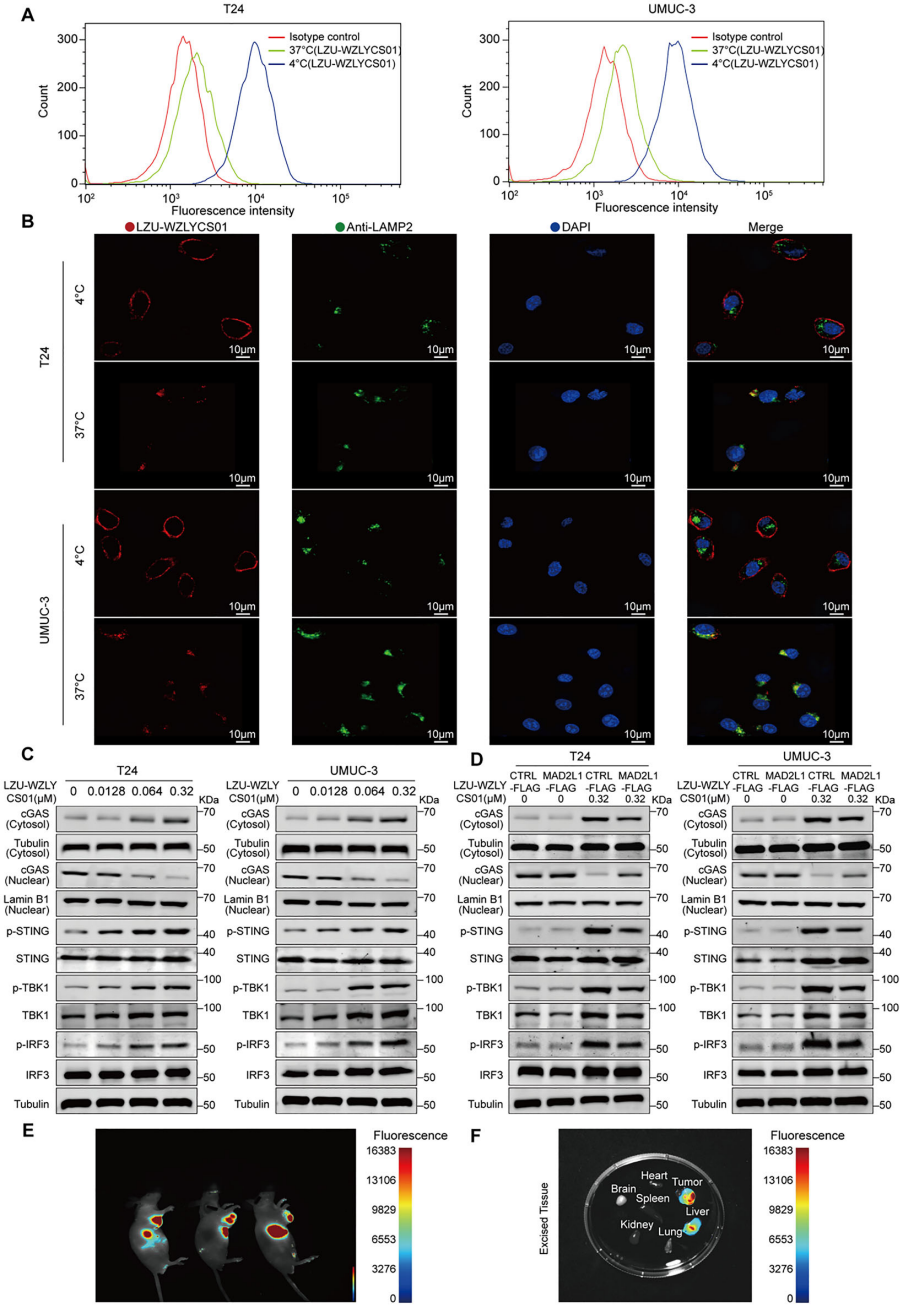
Mechanism of action and tumor‐targeting capability of LZU‐WZLYCS01. A) Flow cytometry analysis of LZU‐WZLYCS01 binding and internalization in T24 and UMUC‐3 cells at 37 and 4 °C. B) Representative images showing LZU‐WZLYCS01 binding, internalization, and lysosomal co‐localization in T24 and UMUC‐3 cells at 37 and 4 °C. Red: LZU‐WZLYCS01, green: anti‐LAMP2, blue: DAPI, scale bar = 10 µm. C) Western blot analysis of cGAS‐STING pathway protein expression in T24 and UMUC‐3 cells treated with different concentrations of LZU‐WZLYCS01 for 48 h. D) Western blot analysis of cGAS‐STING pathway protein expression in MAD2L1‐OE and control cells treated with different concentrations of LZU‐WZLYCS01 for 48 h. E) In vivo fluorescence imaging of UMUC‐3 xenograft models 24 h after intravenous injection of LZU‐WZLYCS01–Cy5 (*n* = 3). F) Ex vivo fluorescence imaging of tumors and major organs (heart, liver, spleen, lungs, kidneys, and brain) collected 24 h post‐injection.

After circulating in the bloodstream, ADCs specifically recognize and bind to tumor cell surface antigens, forming an “ADC–antigen complex.” This complex is internalized via receptor‐mediated endocytosis, forming endosomes that are subsequently transported to lysosomes. Within the lysosomes, ADC is degraded by cathepsins, releasing the cytotoxic payload to kill tumor cells.^[^ ^26^ ^]^ Endocytosis is a critical step for ADCs to enter target cells, and we therefore evaluated the internalization of LZU‐WZLYCS01 in FGFR3‐positive tumor cell lines. Flow cytometry results showed that the fluorescence intensity of LZU‐WZLYCS01 bound to T24 and UMUC‐3 cells at 37 °C was significantly lower than that at 4 °C (where binding is allowed but internalization is inhibited^[^ ^27^ ^]^) (**Figure** **7**A), indirectly indicating that under physiological conditions, LZU‐WZLYCS01 is rapidly internalized into target cells after antigen binding. We further assessed the dynamic trafficking of LZU‐WZLYCS01 in T24 and UMUC‐3 cells using immunofluorescence staining. LZU‐WZLYCS01 (red fluorescence) in the 4 °C group remained localized on the cell surface, while the 37 °C group exhibited typical internalization characteristics: reduced fluorescence intensity on the cell surface and accumulation within lysosomes (green fluorescence) (Figure 7B). We have previously demonstrated that A2 targets MAD2L1 to activate the cGAS‐STING pathway. To investigate whether LZU‐WZLYCS01 operates via the same mechanism, we examined changes in cGAS‐STING pathway‐related proteins in T24 and UMUC‐3 cells treated with varying concentrations of LZU‐WZLYCS01 for 48 h. Western blot results showed that LZU‐WZLYCS01 upregulated cytoplasmic cGAS and increased the protein levels of p‐TBK1, p‐IRF3, and p‐STING (Figure 7C). We then treated MAD2L1‐OE and control cells with LZU‐WZLYCS01 for 48 h and observed changes in these proteins. Western blot analysis revealed that, at the same drug concentration, the protein levels of cytoplasmic cGAS and of p‐TBK1, p‐IRF3, and p‐STING were lower in MAD2L1‐OE T24 and UMUC‐3 cells compared with control cells (Figure 7D). These results suggest that at physiological temperature, LZU‐WZLYCS01 is internalized into cells and transported to lysosomes, where it releases the cytotoxic payload A2, which in turn targets MAD2L1 to activate the cGAS‐STING pathway and induce tumor cell apoptosis. Finally, we evaluated the in vivo tumor‐targeting capability of LZU‐WZLYCS01 using a UMUC‐3 xenograft model. At 24 h after intravenous injection of LZU‐WZLYCS01–Cy5, in vivo fluorescence imaging showed significant fluorescence accumulation in the tumor and liver regions (Figure 7E). Similarly, ex vivo fluorescence imaging of major organs and tumor tissues collected 24 h post‐injection further confirmed these findings: strong fluorescence signals were predominantly localized in tumor tissues and the liver, whereas negligible fluorescence was observed in the heart, spleen, lungs, kidneys, or brain (Figure 7F).

### 2.8 In Vivo Antitumor Activity of LZU‐WZLYCS01

**Figure 8:**
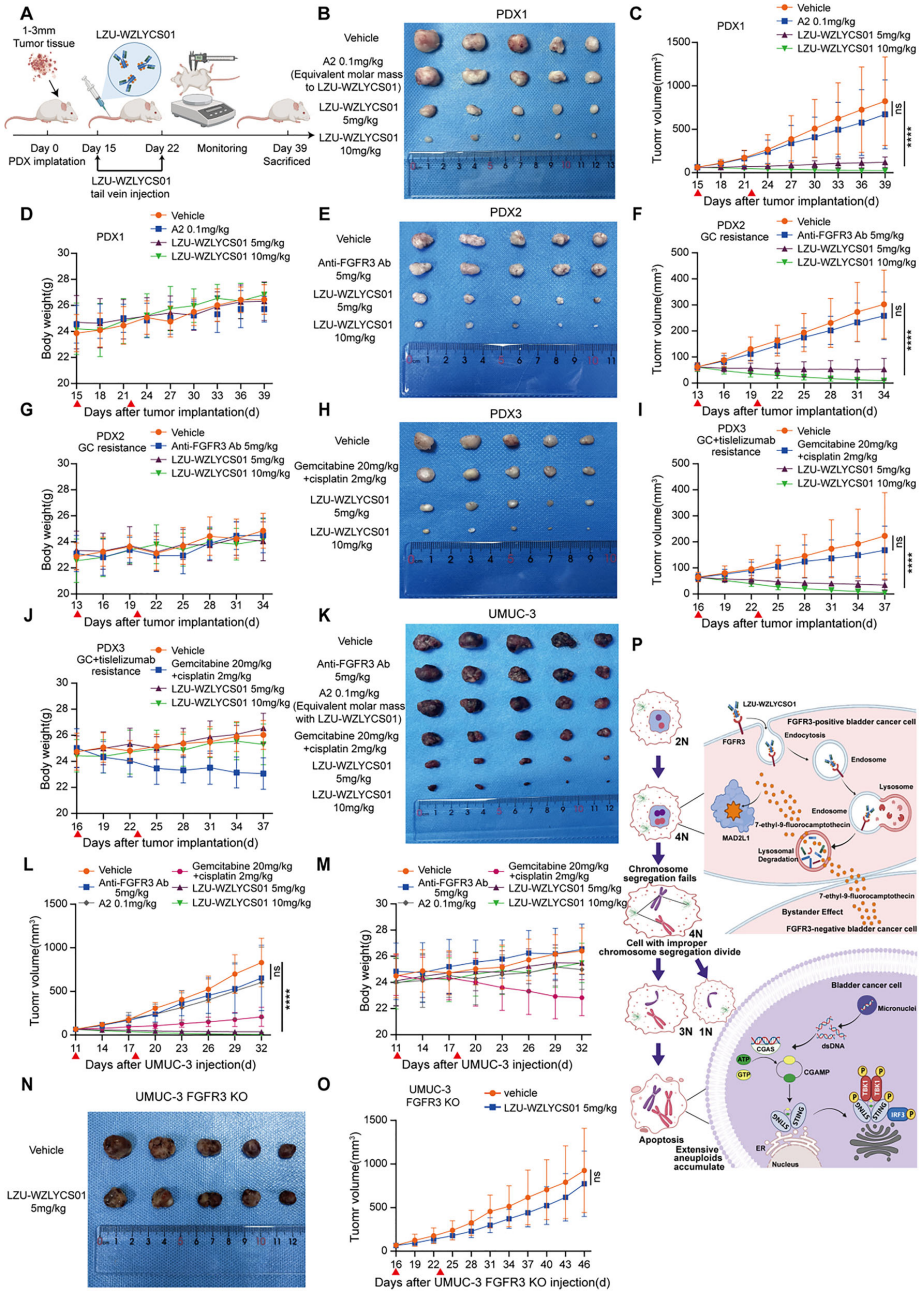
In vivo antitumor activity of LZU‐WZLYCS01. A) Experimental flowchart of LZU‐WZLYCS01 treatment in PDX tumors. B–D) PDX1 tumors, representative images (B), tumor growth curves over time (C), and body weight changes (D) in different treatment groups. E‐G) PDX2 tumors representative images (E), tumor growth curves over time (F), and body weight changes (G) in different treatment groups. H–J) PDX3 tumors representative images (H), tumor growth curves over time (I), and body weight changes (J) in different treatment groups. K–M) UMUC‐3 xenograft tumors representative images (K), tumor growth curves over time (L), and body weight changes (M) in different treatment groups. N,O) UMUC‐3 FGFR3‐KO xenograft tumors, representative images (N) and tumor growth curves over time (O). P) Schematic diagram of the mechanism of action of LZU‐WZLYCS01. LZU‐WZLYCS01 was administered via tail vein injection once weekly for 2 doses (indicated by ▲). Data are presented as the mean ± SD (*n* = 5). ****P < 0.0001. Statistical significance was determined by two‐way ANOVA followed by Tukey's multiple comparison test (C,F,I,L,O).

We evaluated the in vivo antitumor activity of LZU‐WZLYCS01 in UMUC‐3 xenograft and more clinically relevant PDX models. PDX2 and PDX3 were derived from BC tissue samples resistant to GC chemotherapy and dual‐resistant to GC combined with tislelizumab treatment, respectively. IHC results demonstrated positive UPK2 expression in all three PDX tumors (Figure S9, Supporting Information). This finding confirms that the established PDX models retained key histopathological characteristics of urothelial carcinoma. Mice were intravenously injected with 5 or 10 mg kg^−1^ LZU‐WZLYCS01 (once weekly for 2 doses), and tumor volumes were measured every 3 days after treatment initiation (**Figure** **8**A). Results showed that both 5 and 10 mg kg^−1^ LZU‐WZLYCS01 significantly inhibited tumor growth in all three PDX models (Figure 8B,C,E,F,H,I). Next, we assessed the antitumor activity of LZU‐WZLYCS01, GC, A2 (at an equivalent molar mass to LZU‐WZLYCS01), and the anti‐FGFR3 antibody in the UMUC‐3 xenograft models. Neither the anti‐FGFR3 antibody (5 mg kg^−1^) nor A2 (0.1 mg kg^−1^) exhibited significant tumor growth inhibition, and gemcitabine (20 mg kg^−1^) combined with cisplatin (2 mg kg^−1^) showed moderate therapeutic effects. In contrast, 5 and 10 mg kg^−1^ LZU‐WZLYCS01 demonstrated potent antitumor activity (Figure 8K,L). To validate the FGFR3‐dependent antitumor effect of LZU‐WZLYCS01 in vivo, we assessed its activity in UMUC‐3 FGFR3‐KO xenografts. Results showed that 5 mg kg^−1^ LZU‐WZLYCS01 did not significantly inhibit tumor growth (Figure 8N,O).

IHC revealed that LZU‐WZLYCS01 treatment markedly downregulated the expression of FGFR3, MAD2L1, and Ki‐67 in tumor tissues (Figure S10A–C, Supporting Information). To evaluate the safety of LZU‐WZLYCS01, we monitored mouse body weight during treatment and performed H&E staining on collected organ tissues post‐treatment. Unlike GC chemotherapy and A2 treatment, which caused weight loss (Figures 1, 8), LZU‐WZLYCS01 treatment resulted in stable weight gain in both UMUC‐3 xenografts and PDX models (Figure 8D,G,J,M). H&E staining revealed no significant histopathological alterations in organs potentially affected by the drug, including FGFR3‐high‐expressing organs such as the brain, skin, and testes (Figure S10D, Supporting Information). Hematological parameters, liver and kidney function markers, and myocardial function markers remained within physiological ranges after LZU‐WZLYCS01 treatment (Tables S1,S2, Supporting Information). These results indicate that conjugating the highly potent but toxic compound A2 to an FGFR3 antibody via a cleavable linker enables precise tumor targeting. This design significantly enhances therapeutic efficacy while minimizing systemic toxicity, achieving the goal of “precise targeting, enhanced efficacy, and reduced toxicity”.

## 3 Discussion

The treatment of BC remains a significant challenge in clinical practice. In recent years, with the deep integration of high‐throughput sequencing technologies and molecular biology research, the molecular genetic characteristics of BC have been increasingly elucidated, and key targets in the process of tumorigenesis and progression have been precisely identified. Consequently, targeted therapeutic strategies based on precision medicine have emerged as a promising new treatment option for BC patients. By specifically inhibiting critical driver genes in tumor cells, targeted therapy acts more precisely on tumor cells and significantly reduces toxicity to normal tissues compared with conventional chemotherapy.^[^ ^28^ ^]^ Despite these advances, the clinical application of targeted drugs still faces several challenges. First, the mechanism of action of most traditional targeted agents primarily involves modulation of signaling pathways rather than direct cytotoxicity, often resulting in clinical efficacy that fails to significantly surpass chemotherapy.^[^ ^29^, ^30^ ^]^ Second, the high intratumoral and intertumoral heterogeneity of BC leads to highly individualized responses to targeted therapy.^[^ ^31^ ^]^ More importantly, resistance mechanisms such as frequent target mutations and compensatory bypass activation during treatment further limit the long‐term efficacy of targeted drugs.^[^ ^32^ ^]^ ADCs combine the precise targeting of monoclonal antibodies with the potent cytotoxicity of payloads, providing a novel strategy to overcome the therapeutic limitations of traditional targeted drugs in BC.^[^ ^33^ ^]^

In this study, we conjugated A2 to a humanized IgG1 anti‐FGFR3 antibody via a GGFG cleavable linker, successfully constructing a novel ADC drug LZU‐WZLYCS01. To validate LZU‐WZLYCS01's affinity and targeting specificity, we conducted comprehensive in vitro and in vivo studies. ELISA results confirmed that LZU‐WZLYCS01 retained FGFR3‐binding affinity comparable to the parental antibody, indicating that conjugation of A2 to the FGFR3 antibody did not compromise the antibody's biological activity. In FGFR3‐KO cell models and xenograft tumors, LZU‐WZLYCS01's antitumor efficacy was markedly diminished, confirming its FGFR3‐dependent mechanism. For any ADC, a critical consideration is its ability to efficiently accumulate at the tumor site following systemic administration while minimizing off‐target exposure. Our in vivo fluorescence imaging revealed the robust and specific accumulation of LZU‐WZLYCS01–Cy5 in tumor tissues, demonstrating highly efficient tumor targeting.

LZU‐WZLYCS01 demonstrated remarkable antitumor effects across cellular levels, clinically relevant PDOs, and xenograft models (cell line‐derived xenograft (CDX) and PDX). Notably, the anti‐FGFR3 antibody alone exhibited negligible inhibition of tumor growth, confirming that the cytotoxic payload A2 is the core effector molecule responsible for LZU‐WZLYCS01's antitumor activity. Platinum‐based combination chemotherapy remains the current first‐line treatment for advanced or metastatic BC. However, clinical experience shows that many patients develop primary or acquired resistance, limiting its treatment efficacy.^[^ ^34^ ^]^ In this study, we found that in UMUC‐3 xenograft models, LZU‐WZLYCS01 demonstrated superior tumor growth inhibition compared with GC chemotherapy. Notably, LZU‐WZLYCS01 maintained significant antitumor activity even in GC‐resistant PDX models. We hypothesize that this advantage may be attributed to the unique molecular mechanism of the cytotoxic payload A2 within LZU‐WZLYCS01. Regarding safety, LZU‐WZLYCS01 showed superior tolerability compared with A2 and GC chemotherapy. During treatment, mice maintained a steady increase in body weight, with no significant histopathological abnormalities observed in major organs or in liver, kidney, and cardiac function assays. These findings indicate that LZU‐WZLYCS01 possesses exceptional tumor‐targeting capability, which effectively reduces off‐target toxicity and significantly enhances therapeutic safety.

The remarkable antitumor activity of LZU‐WZLYCS01 is closely associated with the selection of FGFR3 as the target and the rational design of the ADC components. As a member of the FGFR family, FGFR3 exhibits highly specific expression in BC, providing an ideal molecular basis for the targeted therapy of LZU‐WZLYCS01. This selective expression pattern significantly enhances tumor‐specific accumulation of the ADC, improving therapeutic efficacy while reducing off‐target toxicity. Furthermore, similar to EGFR/HER2, FGFR3 exhibits robust receptor internalization upon antibody binding, a feature that facilitates efficient intracellular delivery of the cytotoxic payload and enhances the antitumor activity of ADCs.^[^ ^35^ ^]^ After validating FGFR3 as a BC‐specific target for ADC therapy, we employed a humanized IgG1 antibody as the drug carrier to confer an extended plasma half‐life, thereby facilitating adequate tumor penetration of the ADC. The use of a GGFG cleavable linker enables LZU‐WZLYCS01 to be degraded by lysosomal cathepsins. This precise enzyme‐responsive release not only maximizes drug activity within target cells but, more importantly, generates a pronounced bystander effect due to A2's high membrane permeability, further enhancing the ADC's overall therapeutic efficacy.^[^ ^36^ ^]^ While immunogenicity remains a critical concern in ADC development, several design features of LZU‐WZLYCS01 are anticipated to mitigate this risk through synergistic mechanisms. First, the humanized IgG1 antibody not only minimizes the content of non‐human sequences to reduce the potential for eliciting anti‐drug antibodies (ADAs) but also reduces the innate propensity for global protein aggregation through its inherent biophysical stability.^[^ ^26^ ^]^ Second, the GGFG peptide linker confers high plasma stability, which ensures the ADC remains intact during systemic circulation. Additionally, the inherent hydrophilicity of the linker reduces the overall hydrophobicity of the ADC conjugate, thereby reducing its propensity for aggregation.^[^ ^37^ ^]^ Finally, beyond these molecular design features, the superior tumor‐specific targeting demonstrated by our LZU‐WZLYCS01 biodistribution data (Figure 7E,F) is expected to minimize systemic exposure, thereby further reducing the overall immunogenic potential of LZU‐WZLYCS01.

The remarkable antitumor activity of LZU‐WZLYCS01 is further attributed to the potent efficacy and unique molecular mechanism of its cytotoxic payload A2. Camptothecin derivatives have emerged as one of the most promising cytotoxic payloads for ADC development after microtubule inhibitors, owing to their potent antitumor activity. Building upon our previously developed camptothecin derivative A2, this study further demonstrates its potent antitumor efficacy in BC. At the cellular level, A2 exhibited potent cytotoxicity against BC cells. More importantly, in clinically relevant models, including PDOs, CDX, and PDX, A2 also exhibited significant tumor growth inhibition. Camptothecin and its derivatives, known for their unique targeting of Topoisomerase I (TopI), have been approved for treating multiple malignancies.^[^ ^38^ ^]^ However, recent studies have suggested that some camptothecin derivatives, such as FL118, may function a**s **multi‐target agents. Our previous work demonstrated that FL118 binds between H2A.X and coiled DNA, inhibiting DNA damage repair and ultimately inducing apoptosis in BC cells.^[^ ^39^ ^]^ Contrary to conventional views on camptothecin and its derivatives, their classic target, TopI, may play a greater role in FL118‐associated adverse effects than in its antitumor efficacy.^[^ ^40^ ^]^ Notably, our findings reveal that although A2 is structurally a camptothecin derivative, its potent antitumor activity in BC is achieved through specific targeting of MAD2L1.

MAD2L1 is a core component of the SAC, which monitors proper chromosome‐spindle microtubule attachment during cell division and ensures accurate sister chromatid segregation.^[^ ^41^ ^]^ Accumulating evidence indicates that MAD2L1 overexpression is closely associated with tumorigenesis and progression in diverse malignancies, including hepatocellular carcinoma, cholangiocarcinoma, colorectal cancer, prostate cancer, and lung cancer.^[^ ^42^, ^43^ ^]^ Our study identifies A2 as a potent MAD2L1 inhibitor and provides its first comprehensive preclinical evaluation as a targeted therapeutic. While the prior study identified M2I‐1 as a small molecule that inhibits the MAD2L1‐CDC20 interaction in vitro, its characterization was limited to this biochemical activity.^[^ ^44^ ^]^ We not only confirmed the direct binding of A2 to MAD2L1 but also demonstrated its potent tumor‐suppressive effects across cellular models, PDOs, and CDX/PDX models. More importantly, we developed LZU‐WZLYCS01, which addresses critical drug delivery challenges and demonstrates superior tumor‐specific accumulation and efficacy.

The conventional view that cGAS is solely a cytosolic sensor of foreign DNA has been revised by findings showing its predominant nuclear localization and inactivation through chromatin binding.^[^ ^45^, ^46^ ^]^ Cytosolic aberrant DNA serves as a danger signal that triggers the translocation of nuclear cGAS into the cytoplasm, where it binds DNA to activate STING‐dependent signaling. Our findings demonstrate that A2 promotes nucleo‐cytoplasmic translocation of cGAS, activates the cGAS‐STING pathway, upregulates cytoplasmic cGAS protein levels, and enhances the expression of p‐TBK1, p‐IRF3, and p‐STING proteins. Accumulating evidence indicates that the cGAS‐STING pathway not only mediates inflammatory responses and type I interferon (IFN) production but also regulates critical biological processes including apoptosis.^[^ ^47^, ^48^ ^]^ For instance, during mycobacterial infection, aberrant STING activation triggers ER stress, which leads to BAX activation and cytochrome c release, ultimately inducing apoptosis in RAW264.7 cells.^[^ ^49^ ^]^ In our study, we observed significantly reduced A2‐induced apoptosis in BC cells after adding STING inhibitor treatment, indicating that A2 induces apoptosis via activation of the cGAS‐STING pathway.

In this study, we developed LZU‐WZLYCS01, a novel ADC targeting the BC‐specific antigen FGFR3 with A2 as its cytotoxic payload. LZU‐WZLYCS01 specifically recognizes and binds to FGFR3, undergoing receptor‐mediated endocytosis and subsequent lysosomal cathepsin‐mediated cleavage to release A2 within BC cells. A2 activates the cGAS‐STING pathway by targeting MAD2L1, ultimately inducing apoptosis in BC cells. Notably, a fraction of free A2 passively diffuses into the tumor microenvironment, demonstrating a potent bystander effect that effectively eliminates adjacent FGFR3‐negative tumor cells (Figure 8P). These preclinical results highlight LZU‐WZLYCS01's significant potential as a novel therapeutic agent for BC. Its unique target complements existing BC ADCs, not only expanding the scope of ADC applications in BC treatment but also potentially achieving comprehensive coverage of key therapeutic targets. This breakthrough may provide a new strategic option for precision therapy in BC.

## 4 Experimental Section

### Cell Lines and Cell Culture

Two human BC cell lines, UMUC‐3 (RRID: CVCL_1783) and T24 (RRID: CVCL_0554), were purchased from the Cell Bank of Type Culture Collection, Chinese Academy of Sciences (Shanghai, China). The cells were cultured in RPMI‐1640 medium (Gibco, USA) supplemented with 10% fetal bovine serum (FBS) and 1% antibiotics (100 U mL^−1^ penicillin and 100 µg mL^−1^ streptomycin). All cell cultures were maintained at 37 °C in a humidified atmosphere containing 5% CO_2_. The cells were confirmed to be free of mycoplasma contamination in all experiments.

### Cell Viability Assessment with CCK‐8

The cytotoxicity of A2 against T24 and UMUC‐3 cells was evaluated using the CCK‐8 (C0037, Beyotime, China). T24 and UMUC‐3 cells were seeded in 96‐well plates (6 × 10^3^ cells well^−1^ for 48 h, and 4 × 10^3^ cells well^−1^ for 72 h), and cultured for 24 h. The cells were then treated with varying concentrations of A2 (0.00002048, 0.0001024, 0.000512, 0.00256, 0.0128, 0.064, 0.32, 1.6, and 8 µm) for 48 or 72 h, with untreated cells as the control. After discarding the original culture medium, a mixture of 10 µL CCK‐8 reagent and 90 µL complete culture medium was added to each well and incubated for 1 h. The absorbance was measured at 450 nm using a microplate reader.

To investigate the cytotoxicity of LZU‐WZLYCS01 against WT and FGFR3‐KO T24 and UMUC‐3 cells, the cells were seeded in 96‐well plates (4 × 10^3^ cells well^−1^) and cultured for 24 h. Subsequently, the cells were treated with varying concentrations of LZU‐WZLYCS01, A2, and the anti‐FGFR3 antibody (0.00002048, 0.0001024, 0.000512, 0.00256, 0.0128, 0.064, and 0.32 µm) for 72 h. The subsequent experimental procedures were performed as described above.

### Colony Formation Assay

T24 and UMUC‐3 cells were seeded in 6‐well plates (500 cells well^−1^) and cultured for 72 h. The cells were then treated with different concentrations (0.0128 and 0.064 µm) of A2 or LZU‐WZLYCS01 for 48 h, with untreated cells as the control. After replacing the medium with fresh complete medium, the cells were cultured for an additional 7–10 days. After washing with PBS, the colonies were fixed with 4% paraformaldehyde, stained with 0.1% crystal violet for 15 min, and finally photographed and counted. To investigate the effect of A2 on the colony formation of MAD2L1‐OE cells, MAD2L1‐OE T24 and UMUC‐3 cells, along with control cells, were seeded in 6‐well plates (500 cells well^−1^) and cultured for 72 h. The cells were then treated with 0.0128 µm A2 for 48 h, followed by the same experimental procedures as described above.

### Cell Cycle and Aneuploidy Analysis

T24 and UMUC‐3 cells were seeded in 6‐well plates (2.5 × 10⁵ cells well^−1^) and cultured for 24 h. The cells were then treated with different concentrations (0.0128, 0.064, 0.32, and 1.6 µm) of A2 for 24 h, with untreated cells as the control. The cell suspension was collected and fixed overnight at −20 °C with prechilled 70% ethanol. After washing with PBS, the cells were incubated with RNase A at 37 °C for 30 min, then stained with 50 µg mL^−1^ PI (E‐CK‐A351, Elabscience, China) for 30 min. Cell cycle analysis was performed using a flow cytometer (Beckman CytoFLEX, USA). To determine whether A2 induces aneuploidy, T24 and UMUC‐3 cells were synchronized at the G1/S phase using a double thymidine block. The cells were cultured in medium containing 2 mm thymidine (T8080, Solarbio, China) for 18 h, washed and released into fresh complete medium for 8 h, and then re‐treated with 2 mm thymidine for 16 h. After cell cycle synchronization, cells were treated with 0.064 µm A2 for different time periods, harvested, and fixed overnight at −20 °C with prechilled 70% ethanol. The subsequent experimental procedures were performed as described above.

### Apoptosis Analysis

Cells were seeded in 6‐well plates (1.5 × 10⁵ cells well^−1^) and cultured for 24 h. The cells were then treated with different concentrations of A2 (0.0128, 0.064, 0.32, and 1.6 µm), Topotecan (0.0128, 0.064, 0.32, and 1.6 µm), or LZU‐WZLYCS01 (0.0128, 0.064, and 0.32 µm) for 48 h, with untreated cells as the control. The cell suspension was collected and incubated with 5 µL Annexin V‐FITC and 5 µL PI (E‐CK‐A211, Elabscience, China) at room temperature in the dark for 15 min. Apoptosis analysis was performed using a flow cytometer.

To investigate the effect of A2 on apoptosis in MAD2L1‐OE cells, MAD2L1‐OE T24 and UMUC‐3 cells, along with control cells, were seeded in 6‐well plates (1.5 × 10⁵ cells well^−1^) and cultured for 24 h. The cells were then treated with 1.6 µm A2 for 48 h. The same procedure was performed using MAD2L1‐KD cells to assess the effect in the knockdown context. The cell suspension was collected and incubated with either 5 µL Annexin V‐APC and 10 µL PI (AP107, MultiSciences, China) for the MAD2L1‐OE cells, or 5 µL Annexin V‐FITC and 5 µL PI for the MAD2L1‐KD cells, at room temperature in the dark for 15 min. Apoptosis analysis was performed using a flow cytometer.

### Pull‐Down/MS Analysis

To identify potential cellular targets interacting with A2, pull‐down experiments were performed, followed by Western blot and MS analysis. As shown in Figure 2A, the 20th hydroxyl group of A2 was biotinylated to bind streptavidin magnetic beads, enabling the collection of A2‐bound proteins. T24 cell lysates were incubated with A2‐biotin, biotin (negative control), A2‐biotin + 5× molar excess of unlabeled A2, or A2‐biotin + 100× molar excess of unlabeled A2 (competition groups) at 4 °C for at least 2 h, followed by the addition of 100 µL streptavidin magnetic beads (P2151, Beyotime, China) and further incubation overnight at 4 °C. After washing three times with PBS to remove unbound proteins, 80% of the bead mixture was subjected to MS analysis (SpecALLY, China), while the remaining 20% was used for Western blot validation.

To further identify the binding site of A2 on MAD2L1, T24 MAD2L1WT, T24 MAD2L1V55A+I62A+V69A, and T24 MAD2L1K73A cell lysates were incubated with A2‐biotin at 4 °C for at least 2 h, followed by the addition of 100 µL streptavidin magnetic beads (P2151, Beyotime, China) and further incubation overnight at 4 °C. The subsequent experimental procedures were performed as described above.

### CETSA

CETSA was conducted to validate the binding of A2 to MAD2L1, following the protocol described in the literature.^[^ ^50^ ^]^ T24 MAD2L1WT, T24 MAD2L1V55A+I62A+V69A, and T24 MAD2L1K73A cells were cultured in 100 mm dishes until 80%–90% confluency, then treated with 0.32 µm A2 for 2 h at 37 °C. After two PBS washes, cells were resuspended in PBS containing PMSF and aliquoted equally into PCR tubes. Samples were heated at graded temperatures (40, 42.8, 45.8, 49.2, 53.8, 55, 58, 59.8, 63.5, and 67 °C) for 3 min using a thermal cycler. After incubation at room temperature for 3 min, the samples were rapidly frozen in liquid nitrogen for 3 min and thawed at room temperature. Following a second freeze–thaw cycle, the cell lysates were centrifuged at 12 000 × g for 15 min at 4 °C, and the supernatants were collected. Western blot was performed after heat denaturation at 70 °C for 10 min.

### SPR

SPR experiments were performed using a CM5 chip with amine coupling to immobilize MAD2L1 protein (HY‐P71540, MCE, USA). The chip surface was activated with 1‐ethyl‐3‐(3‐dimethylaminopropyl) carbodiimide (EDC) and N‐hydroxysuccinimide (NHS), followed by the conjugation of MAD2L1 at a flow rate of 10 µL min^−1^. The chip was then blocked with ethanolamine.

### Immunofluorescence Staining

T24 cells were treated with 0.32 µm A2‐biotin for 3 h, fixed with 4% paraformaldehyde, permeabilized with 0.5% Triton X‐100, and blocked with 5% BSA for 1 h at room temperature. The cells were incubated with an anti‐MAD2L1 antibody (10337‐1‐AP, Proteintech) and streptavidin‐AF594 (K1068R‐AF594, Solarbio, China) overnight at 4 °C. Subsequently, a CL647‐conjugated secondary antibody against MAD2L1 (SA00014‐9, Proteintech) was added and incubated for 1 h at room temperature in the dark. Nuclei were counterstained with Hoechst 33342 (C0031, Solarbio, China), and images were acquired using a confocal microscope (Zeiss LSM880, Germany).

To investigate whether A2 induces micronuclei formation and cGAS translocation to the cytoplasm, T24 and UMUC‐3 cells were treated with 0.064 µm A2 for 48 h. After fixation, permeabilization, and blocking, the cells were incubated overnight at 4 °C with an anti‐cGAS antibody (15 102 s, Cell Signaling Technology). Subsequently, a CL647‐conjugated secondary antibody against cGAS (SA00014‐9, Proteintech) was added and incubated for 1 h at room temperature in the dark. The cells were then stained with 3 µL mL^−1^ PicoGreen at 37 °C for 1 h, and images were acquired using a confocal microscope.

### Quantification of Cytosolic DNA

Cytosolic DNA was extracted as described in the reference.^[^ ^51^ ^]^ T24 and UMUC‐3 cells were treated with 0.32 µm A2 for 48 h, with untreated cells as the control. Cells were harvested and equally divided; one portion for genomic DNA extraction and the other for cytosolic DNA isolation. For cytosolic DNA, cells were incubated in permeabilization buffer (150 mm NaCl, 2 mm EDTA, 50 mm HEPES (pH 7.4), and 50 µg mL^−1^ digitonin (HY‐N4000, MCE, USA)) for 10 min, followed by centrifugation at 17 000 × g for 10 min. Both genomic and cytosolic DNA were extracted via phenol‐chloroform and precipitated with ethanol. Genomic DNA served as an internal control. qPCR was performed using human 18S‐specific primers: 18S‐Forward: 5′‐GAGGATGAGGTGGAACGTGT‐3′; 18S‐Reverse: 5′‐AGAAGTGACGCAGCCCTCTA‐3′.

### Lentiviral Infection

MAD2L1‐OE and control lentiviruses were purchased from Hanbio Co., Ltd. (Shanghai, China), and MAD2L1‐KD with its control lentiviruses was obtained from Tsingke Co., Ltd. (Beijing, China). MAD2L1WT and mutant lentiviruses (MAD2L1V55A+I62A+V69A, MAD2L1K73A) were obtained from Tsingke Co., Ltd. (Beijing, China). T24 and UMUC‐3 cells were infected with lentiviruses for 24 h in the presence of 5 µg mL^−1^ polybrene (C0351, Beyotime, China). Subsequently, cells were selected using medium containing 2 µg mL^−1^ puromycin (ST551, Beyotime, China). Infection efficiency was confirmed by Western blot.

FGFR3‐KO lentiviruses were obtained from Tsingke Co., Ltd. (Beijing, China). The single guide RNA sequence for FGFR3 was 5′‐CCCACCAGGACACGCUCCGA‐3′. T24 and UMUC‐3 cells were infected with FGFR3‐KO lentiviruses for 24 h in the presence of 5 µg mL^−1^ polybrene, followed by selection with 2 µg mL^−1^ puromycin. Single‐cell clones were isolated, and positive clones were expanded after validation by DNA sequencing and Western blot.

### Preparation and Quality Control of FGFR3 Antibody

Through systematic evaluation of existing FGFR3 monoclonal antibodies (including target affinity and immunogenicity), the recombinant humanized IgG1 FGFR3 monoclonal antibody LY3076226^[^ ^24^ ^]^ was selected for further experiments. The expression plasmid was constructed based on the FGFR3 monoclonal antibody sequence and transfected into CHO cells for recombinant expression. Subsequently, the following methods were employed for antibody quality control: SEC‐HPLC was used to assess the purity of the antibody. Molecular weight and light/heavy chain ratios were verified by SDS‐PAGE under both reducing and non‐reducing conditions, with IPI serving as a reference antibody for structural integrity validation. The antibody's specific binding affinity to the FGFR3 antigen was quantitatively determined by ELISA. Endotoxin levels in antibody samples were quantified by the LAL assay.

### Design and Synthesis of LZU‐WZLYCS01

The antibody was conjugated to the LP via thiol‐based coupling. Briefly, the antibody was buffer‐exchanged into PBS containing 5 mm EDTA using a 30 kDa ultrafiltration device, followed by disulfide bond reduction with tris(2‐carboxyethyl)phosphine (TCEP) at 37 °C for 2 h. After the reaction was completed, 15‐fold molar equivalents of LP were added to the system for coupling at 4 °C. Unreacted LP was removed by 30 kDa ultrafiltration, and LZU‐WZLYCS01 was buffer‐exchanged into a 100 mm proline solution for storage at −80 °C. Following conjugation, the concentration of LZU‐WZLYCS01 was determined by UV–vis spectroscopy. The DAR was analyzed using HIC, while SEC‐HPLC assessed the purity of LZU‐WZLYCS01. The specific binding capacity of LZU‐WZLYCS01 to the FGFR3 antigen was quantitatively measured by ELISA, and endotoxin levels were monitored via the LAL assay.

### Analysis of Cell Surface FGFR3 Expression and LZU‐WZLYCS01 Binding by Flow Cytometry

To evaluate FGFR3 cell surface expression, WT and FGFR3‐KO T24 and UMUC‐3 cells (1 × 10⁶ cells line^−1^) were suspended in FACS buffer and incubated with 10 µg mL^−1^ FGFR3‐AF647 (sc‐390423 AF647, Santa Cruz, USA) at 4 °C for 2 h in the dark. After PBS washing, fluorescence signals were analyzed by flow cytometry.

For evaluation of LZU‐WZLYCS01 binding to cell surface FGFR3 antigen, WT and FGFR3‐KO T24 and UMUC‐3 cells (1 × 10⁶ cells line^−1^) were similarly processed using 10 µg mL^−1^ LZU‐WZLYCS01‐AF647, followed by flow cytometric analysis.

### Protein Extraction and Western Blot

Cells were lysed in ice‐cold RIPA buffer (R0010, Solarbio, China) supplemented with phosphatase inhibitor cocktail (P1260, Solarbio, China) and PMSF (GRF101, Epizyme, China). Lysates were centrifuged at 12 000 × g for 15 min, denatured by boiling, and separated by SDS–PAGE on 7.5%, 10%, or 12.5% gels, followed by transfer onto PVDF membranes. Antibodies Against Caspase‐3 (Ab32351, Abcam), PARP‐1 (13371‐1‐AP, Proteintech), Bax (50599‐2‐Ig, Proteintech), Bcl‐2 (Ab32124, Abcam), MAD2L1 (10337‐1‐AP, Proteintech), Cgas (26416‐1‐AP, Proteintech), Lamin B1 (12987‐1‐AP, Proteintech), Phospho‐STING‐Ser366 (E9A9K, Cell Signaling Technology), STING (66680‐1‐Ig, Proteintech), Phospho‐TBK1‐Ser172 (D52C2, Cell Signaling Technology), TBK1 (67211‐1‐Ig, Proteintech), Phospho‐IRF3‐Ser386 (Ab76493, Abcam), IRF3 (11312‐1‐AP, Proteintech), FGFR3 (HY‐P80399, Medchemexpress), and Β‐Tubulin (66240‐1‐Ig, Proteintech) were diluted according to the instructions and incubated with the PVDF membranes at 4 °C overnight. After washing, membranes were incubated with 1:10 000 diluted fluorescence‐labeled secondary antibodies (Licor, USA) at 37 °C for 1 h in the dark. Image acquisition and data analysis were performed using the Odyssey CLX near‐infrared two‐color fluorescence imaging system (Licor, USA).

### Evaluation of the Bystander Effect

The bystander effect of LZU‐WZLYCS01 was assessed using the co‐culture model depicted in Figure 6D. FGFR3‐KO cells (1 × 10⁵ cells well^−1^) were seeded in 6‐well plates with or without WT cells (1 × 10⁵ cells well^−1^) in the upper chamber. After 24 h of co‐culture, the experiment was divided into three groups: control, LZU‐WZLYCS01 (0.0128 µm), and LZU‐WZLYCS01 (0.0128 µm) co‐cultured with WT cells. After 72 h of incubation, the viability of FGFR3‐KO cells in the lower chamber was quantified by measuring the absorbance at 450 nm using CCK‐8.

### Binding, Internalization, and Lysosomal Co‐Localization Analysis of LZU‐WZLYCS01

The binding and internalization of LZU‐WZLYCS01 were evaluated by flow cytometry. T24 and UMUC‐3 cells (1 × 10⁶ cells line^−1^) were suspended in FACS buffer and incubated with 10 µg mL^−1^ LZU‐WZLYCS01 at 4 °C for 1 h. Cells were then divided into two groups: one continued incubation at 4 °C for 2 h, while the other was shifted to 37 °C for 2 h to assess internalization. After PBS washing, cells were incubated with an FITC‐conjugated goat anti‐human secondary antibody (SA00003‐12, Proteintech) at 4 °C for 1 h in the dark, followed by flow cytometric analysis.

For confocal microscopy analysis of binding, internalization, and lysosomal co‐localization of LZU‐WZLYCS01, T24, and UMUC‐3 cells were seeded in 15 mm glass‐bottom confocal dishes (1.5 × 10⁵ cells well^−1^) and cultured for 24 h. Cells were incubated with 10 µg mL^−1^ LZU‐WZLYCS01–Cy5 at 4 °C for 1 h, then divided into two groups: one continued incubation at 4 °C for 6 h, and the other shifted to 37 °C for 6 h. The cells were fixed with 4% paraformaldehyde, permeabilized with 0.5% Triton X‐100, blocked with 5% BSA for 1 h at room temperature, and then incubated overnight at 4 °C with LAMP2‐CL594 (CL594‐65053, Proteintech). Nuclei were counterstained with Hoechst 33342 (C0031, Solarbio, China), and images were acquired using confocal microscopy.

### Construction of PDOs and A2/LZU‐WZLYCS01‐Mediated Cytotoxicity

All clinical BC tissue samples used in this study were obtained with approval from the Ethics Committee of the Second Hospital of Lanzhou University (Approval No. 2024A‐1319) and with informed consent from all patients. Samples were collected under sterile conditions by trained surgeons after ensuring that clinical diagnostic requirements had been fully met.

BC PDOs were established as described in the literature.^[^ ^52^ ^]^ Fresh tumor tissues were transported on ice, washed, minced, and digested with collagenase/hyaluronidase at 37 °C. After centrifugation at 350 × g, the tissues were further digested with TrypLE Express (Gibco, USA) and then filtered through 100 µm cell strainers. The cell pellet was resuspended in 70% Matrigel (0 827 555, Mogengel, China), gently mixed, and seeded in 24‐well plates at 20–30 µL well^−1^. After incubation at 37 °C with 5% CO_2_ for 30 min to allow Matrigel solidification, 1.5 mL of organoid culture medium (DMEM‐F12 supplemented with 10 ng mL^−1^ epidermal growth factor (EGF), 5% FBS, 10 µm Y‐27632, and 1% penicillin/streptomycin/amphotericin B) was carefully added to each well. The medium was replaced every 3 days to maintain organoid growth.

For drug testing, stable PDOs were treated with A2 (0.064 and 1.6 µm) or LZU‐WZLYCS01 (0.064 and 0.32 µm) for 5 days. Morphological changes were monitored by inverted phase‐contrast microscopy, and drug‐induced cytotoxicity was assessed using Calcein‐AM/PI staining.

### Construction of BC PDX Models and Evaluation of In Vivo Antitumor Activity and Biosafety of A2 and LZU‐WZLYCS01

All animal experiments strictly followed the ethical guidelines outlined in the NIH Guide for the Care and Use of Laboratory Animals, ensuring humane care and treatment for all animals throughout the study. The experimental protocol was approved by the Institutional Animal Care and Use Committee of the Second Hospital of Lanzhou University (Approval No. D2024‐947).

Fresh BC tissues were transported on ice under sterile conditions to the Animal Experiment Center of Lanzhou University. Tumor tissues were dissected into 3 × 3 × 3 mm fragments, mixed with Matrigel, and subcutaneously implanted into the flanks of 4‐ to 5‐week‐old severely immunodeficient NCG mice (NOD/ShiLtJGpt‐Prkdcem26Cd52Il2rgem26Cd22/Gpt, Gempharmatech, China) to establish first‐generation (G1) PDX models. When tumor volumes reached ≈1000 mm^3^, the tumors were excised and passaged into new NCG mice to generate second‐generation (G2) PDX models. Third‐generation (G3) PDX models were used for drug efficacy evaluation. When tumor volumes reached 50–100 mm^3^, mice (*n* = 5 per group) were intraperitoneally administered A2 at 2.5 mg kg^−1^ (100 µL dose^−1^, once weekly for 3 doses). To assess the antitumor activity of LZU‐WZLYCS01, the following treatments were administered via tail vein injection (*n *= 5 per group): anti‐FGFR3 antibody (5 mg kg^−1^), A2 (0.1 mg kg^−1^), gemcitabine (20 mg kg^−1^) + cisplatin (2 mg kg^−1^), LZU‐WZLYCS01 (5 mg kg^−1^), and LZU‐WZLYCS01 (10 mg kg^−1^) (200 µL dose^−1^, once weekly for 2 doses). Body weight and tumor volume were recorded every 3 days. Tumor volume was calculated using the formula: *V* = 0.5 × length × width^2^. At the experimental endpoint, mice were euthanized, and tumors were excised and photographed. Blood samples (0.5 mL) were collected via retro‐orbital bleeding for hematological and biochemical analyses. Major organs (heart, liver, spleen, lungs, kidneys, brain, small intestine, bladder, skin, and testes) were fixed in 4% paraformaldehyde, dehydrated, embedded, sectioned, and subjected to H&E staining to evaluate the biosafety of A2 and LZU‐WZLYCS01. Tumor specimens were analyzed by IHC to evaluate the expression of key proteins (UPK2, FGFR3, MAD2L1, and Ki‐67).

### Construction of CDX Models and Evaluation of In Vivo Antitumor Activity of A2 and LZU‐WZLYCS01

UMUC‐3 cells (1 × 10⁶) were resuspended in PBS, mixed with Matrigel, and subcutaneously injected into the flanks of 4‐ to 5‐week‐old BALB/c nude mice (BALB/cNj‐Foxn1nu/Gpt, Gempharmatech, China). When tumor volumes reached 50–100 mm^3^, treatments were administered following the same regimen as in the PDX model.

To validate the FGFR3‐dependent antitumor effect of LZU‐WZLYCS01 in vivo, UMUC‐3 FGFR3‐KO cells (1 × 10⁶) were subcutaneously injected into the flanks of BALB/c nude mice. When tumors reached 50–100 mm^3^, mice (*n* = 5 per group) received LZU‐WZLYCS01 at 5 mg kg^−1^ (200 µL dose^−1^, once weekly for 2 doses) via tail vein injection. Subsequent procedures were performed as described for the PDX model.

### In Vivo Fluorescence Imaging of LZU‐WZLYCS01–Cy5

The in vivo tumor‐targeting capability of LZU‐WZLYCS01–Cy5 was evaluated using UMUC‐3 xenograft models. When tumors reached 300–400 mm^3^, mice (*n* = 3) were administered LZU‐WZLYCS01–Cy5 at 5 mg kg^−1^ via tail vein injection. At 24 h post‐injection, the biodistribution of LZU‐WZLYCS01–Cy5 was assessed using a DPM wide‐spectrum small animal in vivo optical imaging system (DPM, China). Subsequently, mice were euthanized, and the organ‐specific distribution of LZU‐WZLYCS01–Cy5 was evaluated ex vivo using the DPM system. Fluorescence intensity was recorded in major organs (heart, liver, spleen, lungs, kidneys, brain, and tumors).

### Statistical Analysis

Statistical analyses were performed using GraphPad Prism 9 or SPSS 25.0 software. The association between MAD2L1 expression and OS was evaluated by Cox proportional hazards regression analysis. For comparisons between two groups, a two‐tailed Student's *t*‐test was used, while one‐way or two‐way ANOVA followed by Tukey's multiple comparison test was applied for multi‐group comparisons. Data were presented as mean ± standard deviation (SD). The number of tested samples (*n*) is indicated in the corresponding figures or figure legends, where applicable. A *P*‐value < 0.05 was considered statistically significant. *P < 0.05, **P < 0.01, ***P < 0.001, ****P < 0.0001.

## Conflict of Interest

The authors declare no conflict of interest.

## Author Contributions

S.C., X.F.L., and G.R.F. contributed equally to this work. Z.P.W., Y.Q.L., and S.C. conceptualized the study, participated in the overall design, and supervised and coordinated the research. S.C., X.F.L., and G.R.F. designed and performed the majority of the experiments. J.Q.J., Y.R.W., E.G.Y., J.P.M., and Z.Z. contributed to molecular and cellular biology experiments. Y.H.W., J.W., D.T.W., J.Q.T., and Z.L.D. conducted the animal studies. H.Z.W. performed a pathological analysis. S.C. and X.F.L. wrote the manuscript. Z.P.W. and L.C. reviewed the manuscript. All authors read and approved the final manuscript.

## Supporting information



Supporting Information

## Data Availability

The data that support the findings of this study are available from the corresponding author upon reasonable request.
