# Supplementary material for: A Novel FGFR3‐Targeting Antibody‐Drug Conjugate Induces Tumor Cell Apoptosis through the cGAS–STING Pathway in Bladder Cancer
Source: Adv Sci (Weinh). 2025 Oct 30;13(4):e09933. doi: 10.1002/advs.202509933 (PMC12822473; doi:10.1002/advs.202509933)
Supplement: Supplementary file 1 — Supporting Information [file ADVS-13-e09933-s001.docx]

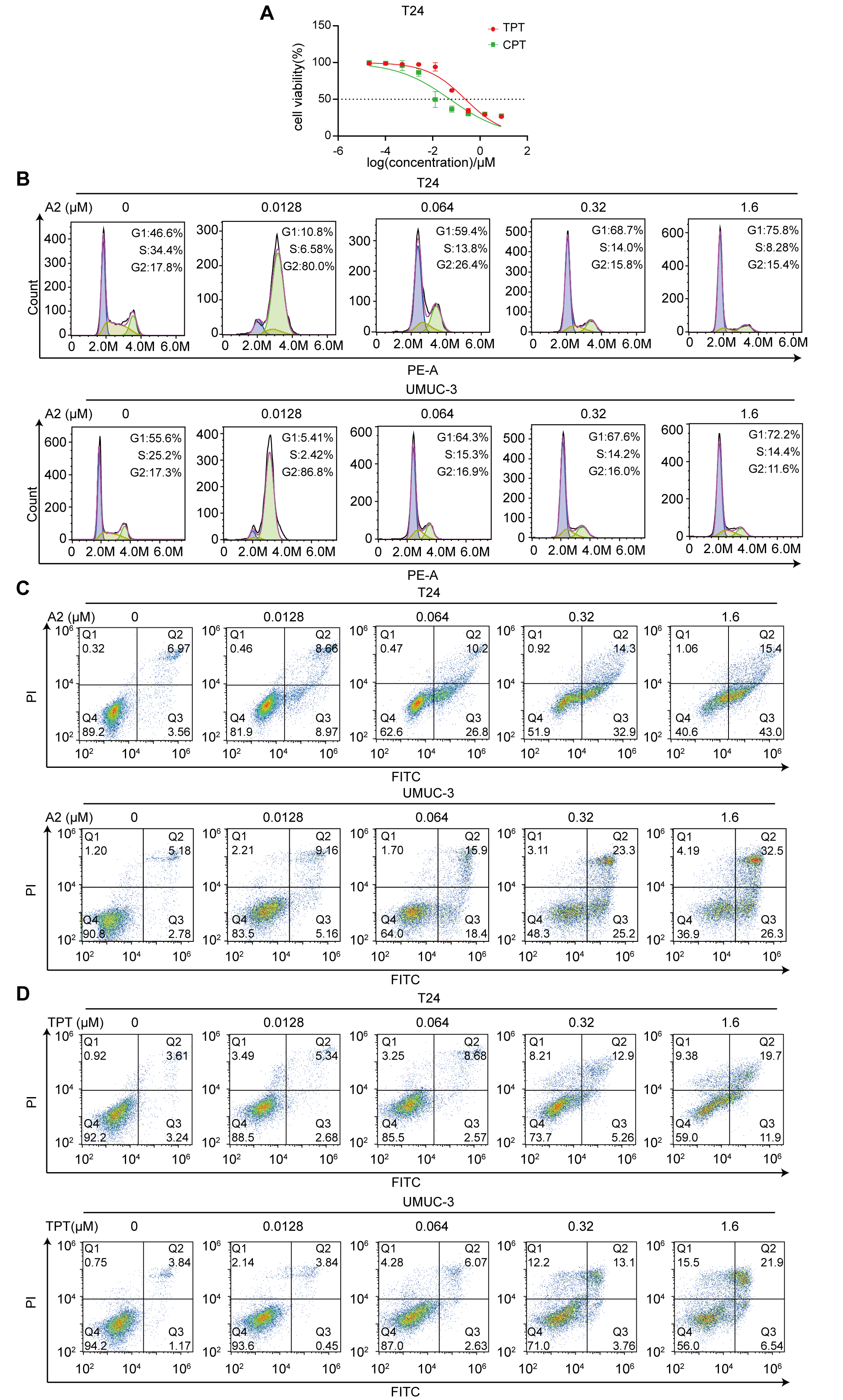
**Figure S1. **Effects of A2, TPT, and CPT on cell viability, cell cycle** distribution**, and apoptosis in** T24 and UMUC-3 cells. A**)**** Cell viability of T24 cells treated with different concentrations of TPT and CPT for 72 h. **B**)**** Cell cycle distribution in T24 and UMUC-3 cells treated with different concentrations of A2 for 24 h. **C**)**** **Apoptotic effects** in T24 and UMUC-3 cells treated with different concentrations of A2 for 48 h. **D**)**** **Apoptotic effects** in T24 and UMUC-3 cells treated with different concentrations of TPT for 48 h.


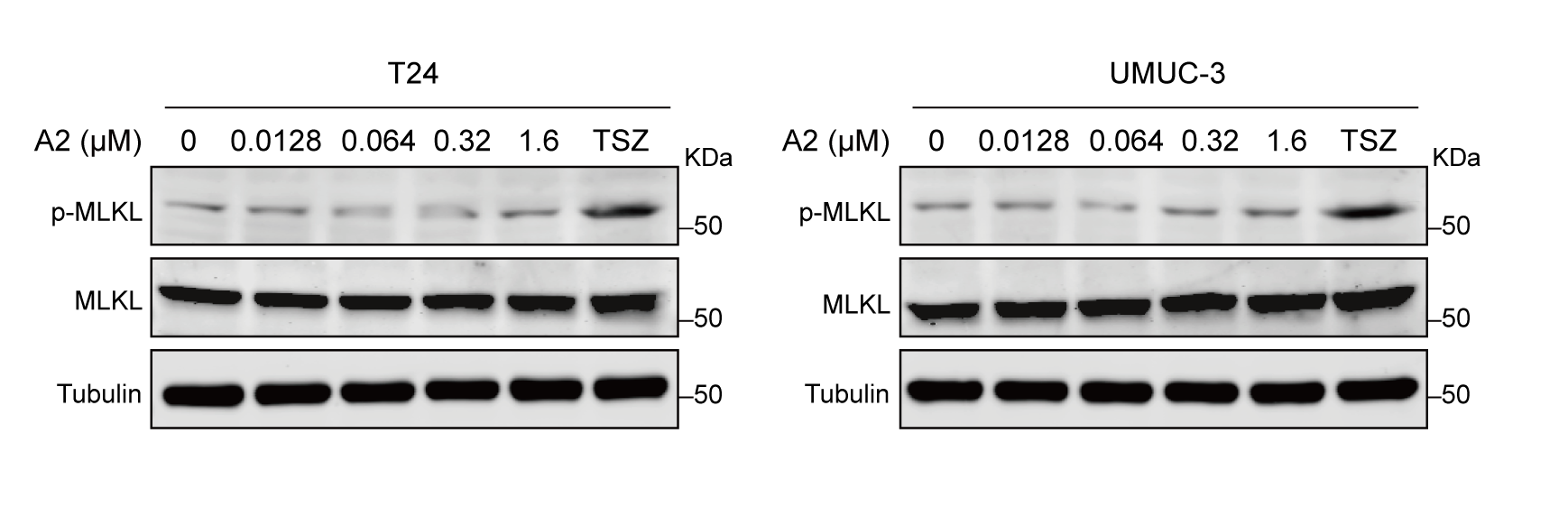


**Figure S2. **Western blot analysis of necroptosis-related proteins in T24 and UMUC-3 cells treated with** different **concentrations of A2 or TSZ**.**


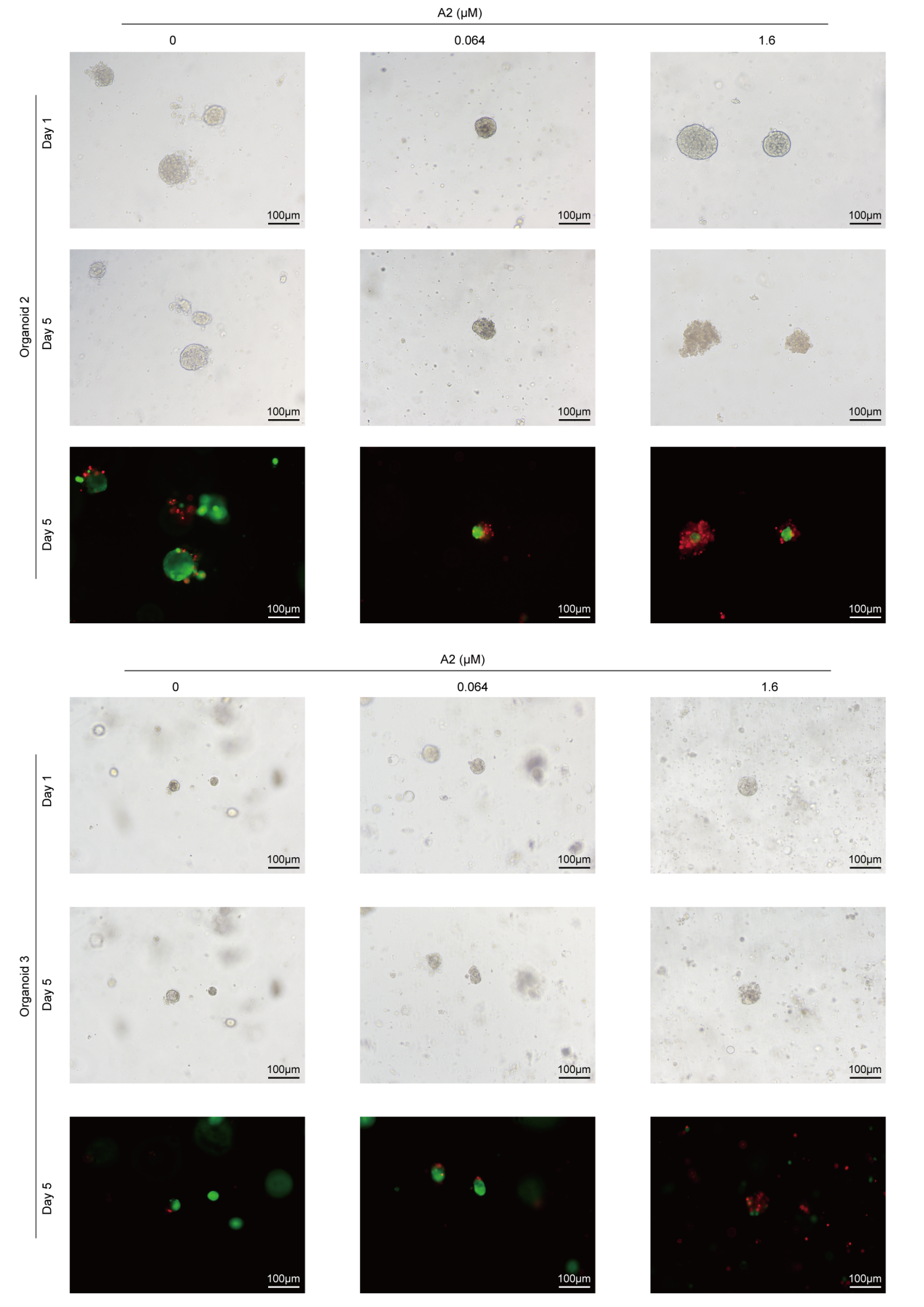


**Figure S3. **Cytotoxicity** of A2 on PDOs.** Representative bright-field and AM/PI-stained images showing A2-induced cytotoxicity in PDOs. Green: viable cells, red: dead cells, scale bar = 100 μm.


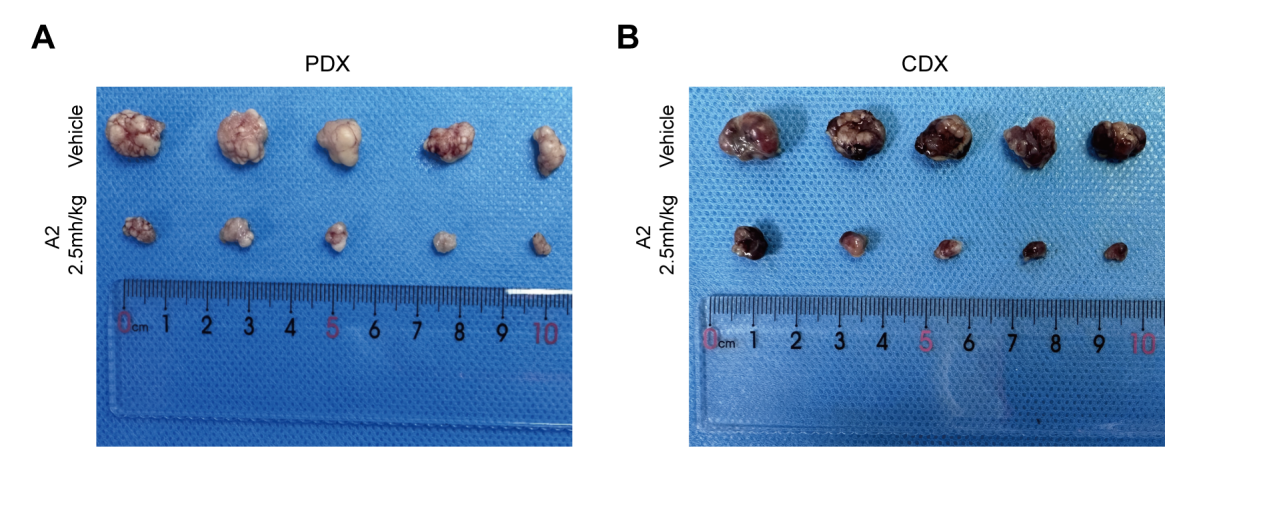


**Figure S4. Representative images of PDX and UMUC-3 xenograft tumors.** ****A**)** PDX tumors. **B)** UMUC-3 xenograft tumors.


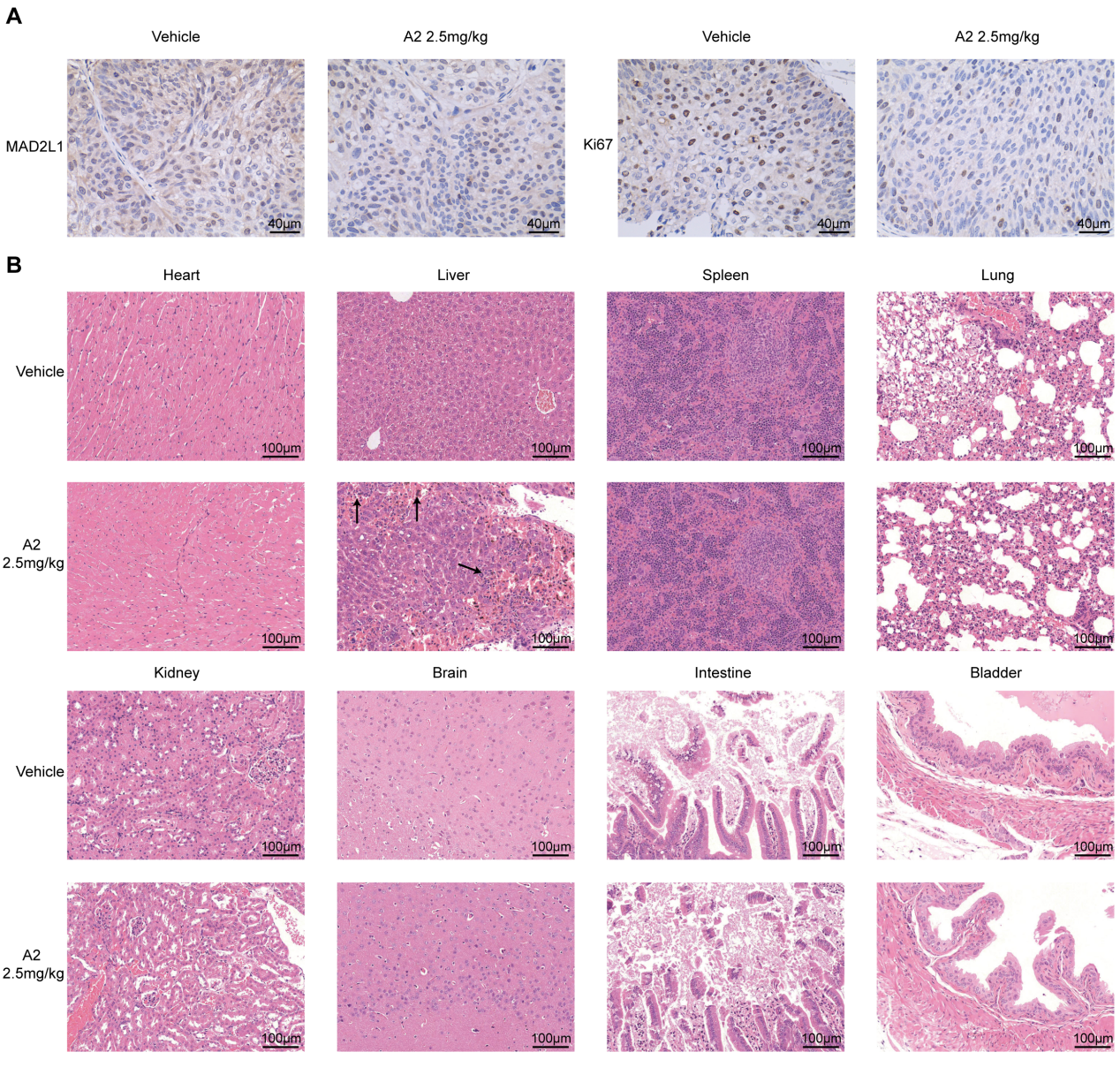


**Figure S5. IHC analysis of tumor tissues and histopathological evaluation of major organs following A2 treatment. A)** Representative IHC staining of MAD2L1 and Ki-67 in PDX tumor tissues. scale bar = 40 μm. **B)** Representative H&E staining of major organs (heart, liver, spleen, lung, kidney, brain, intestine, and bladder). Arrows indicate typical pathological changes in the liver, scale bar = 100 μm.


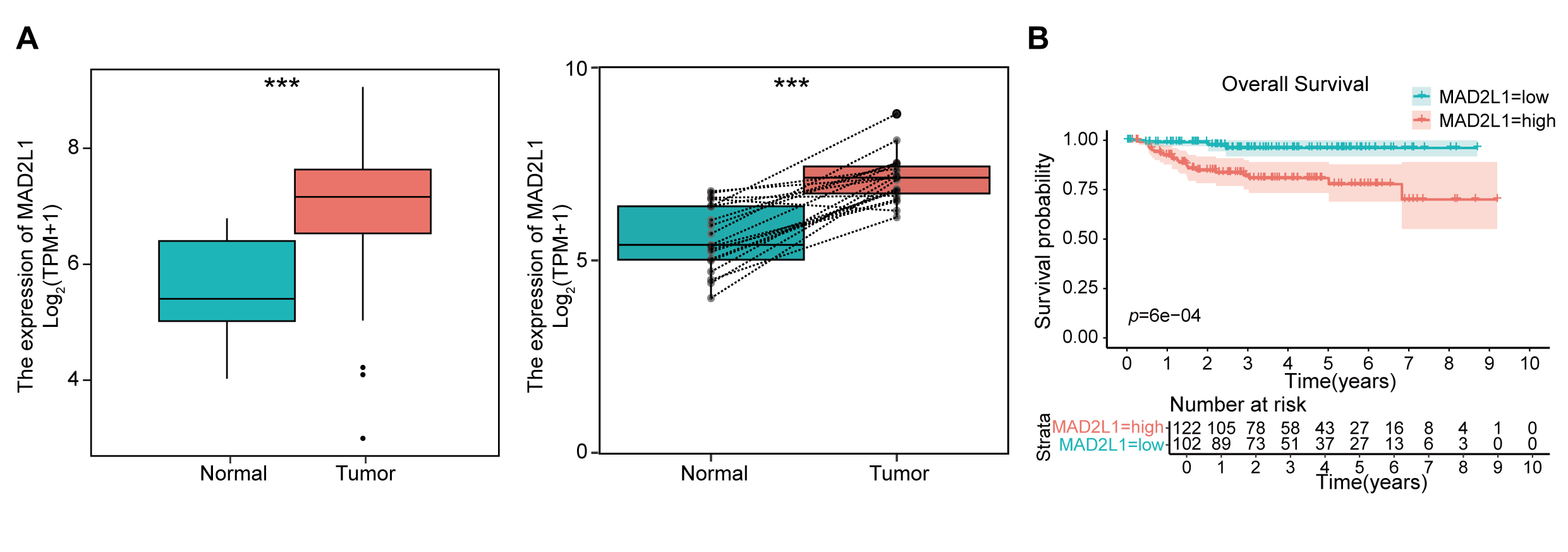


**Figure S6. Expression profile and prognostic significance of MAD2L1 in BC. A)** Transcriptomic analysis of MAD2L1 expression in BC compared to adjacent normal tissues from TCGA database. **B)** Kaplan-Meier survival analysis demonstrating the significant association between MAD2L1 expression and OS in BC patients from GEO database (*P* = 6 × 10⁻⁴). Data are presented as mean ± SD. ***P < 0.001.


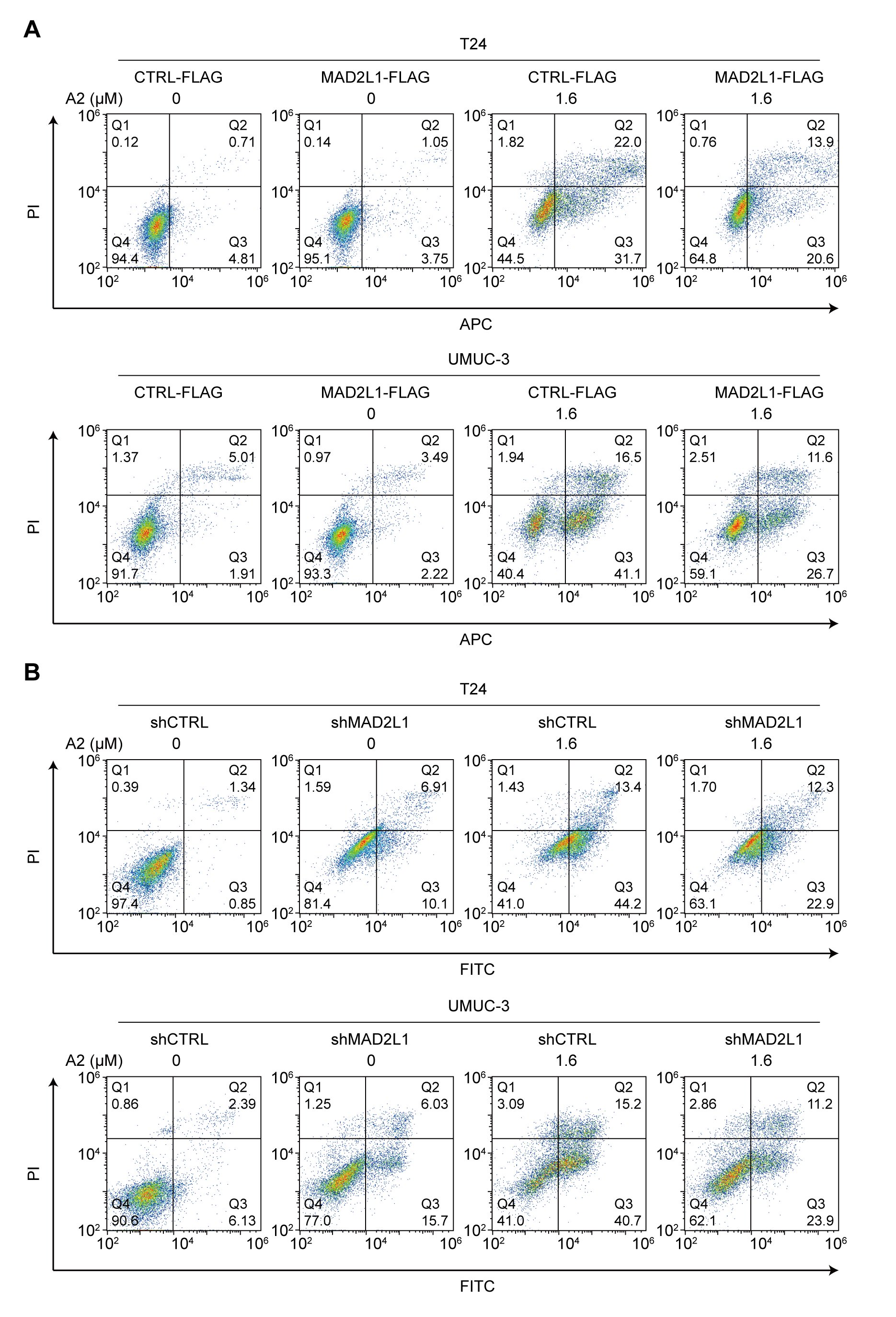
****Figure S7. Apoptotic effects of A2** in MAD2L1-OE and MAD2L1-KD cells **and their controls** A) Apoptotic effects** in MAD2L1-OE and control cells treated with different concentrations of A2 for 48 h. **B**)** Apoptotic effects** in MAD2L1-KD and control cells treated with different concentrations of A2 for 48 h.


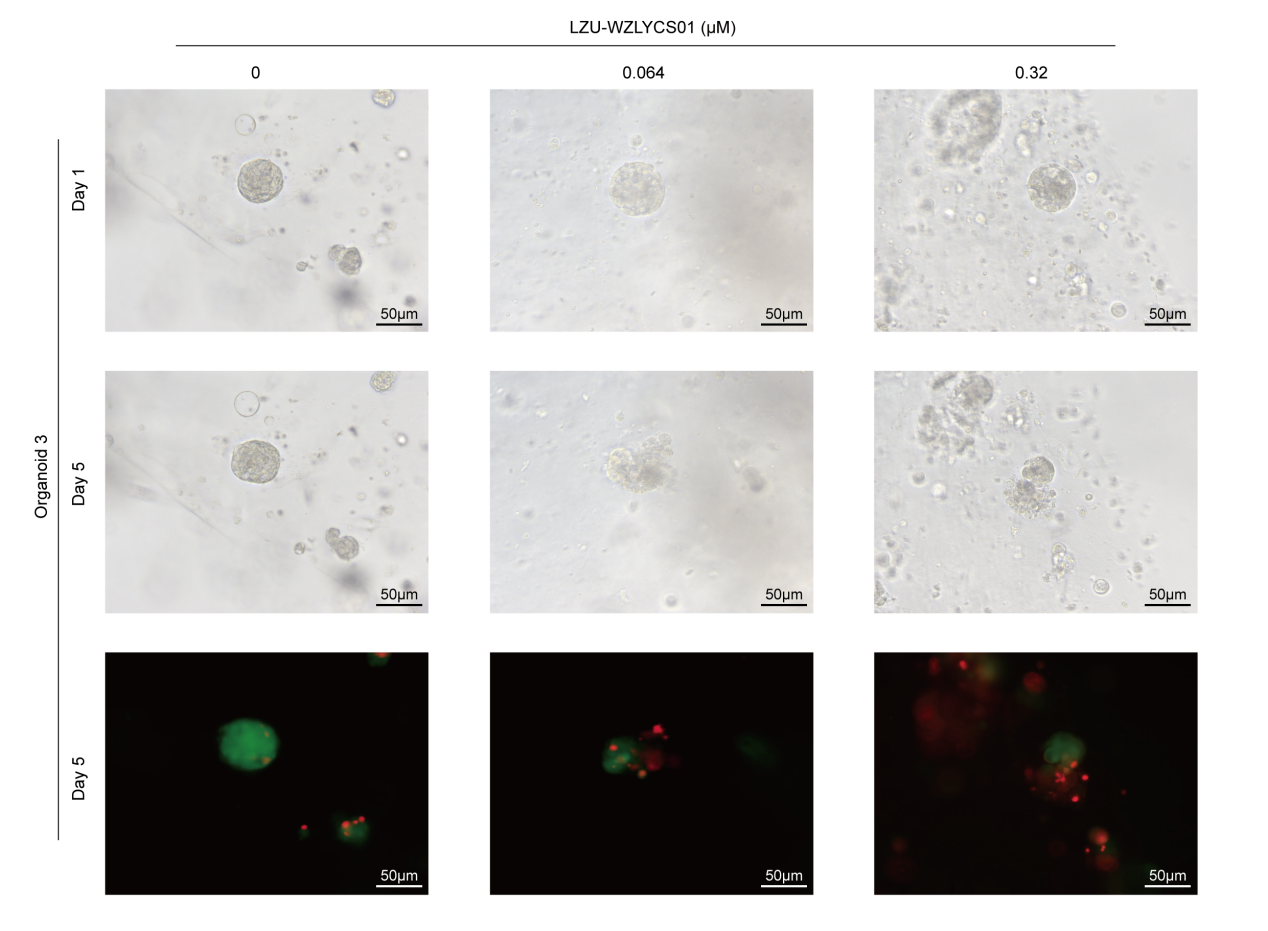


**Figure S8. **Cytotoxicity** of LZU-WZLYCS01 on PDOs.** Representative bright-field and AM/PI-stained images showing LZU-WZLYCS01-induced cytotoxicity in PDOs. Green: viable cells, red: dead cells, scale bar = 50 μm.


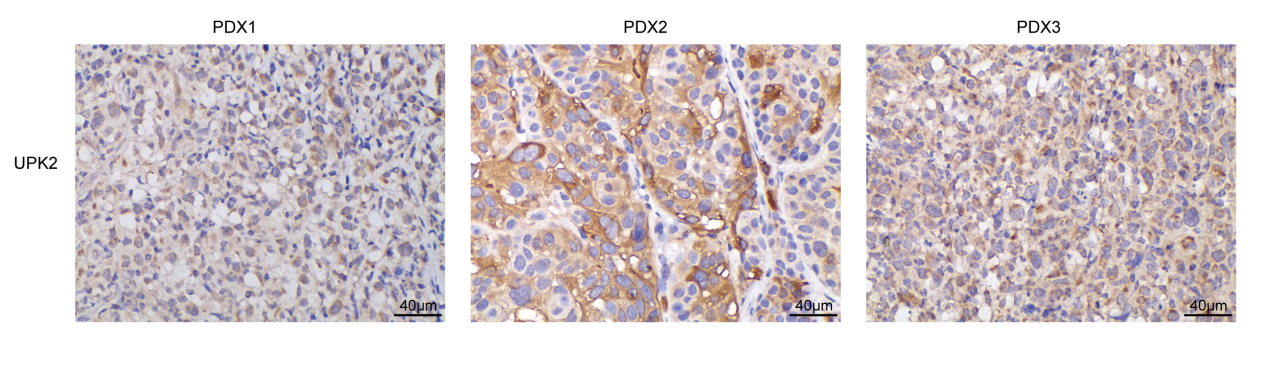
**Figure S9. Representative IHC staining of **UPK2** in **PDX1, PDX2, and PDX3** tumors.** Scale bar = 40 μm.


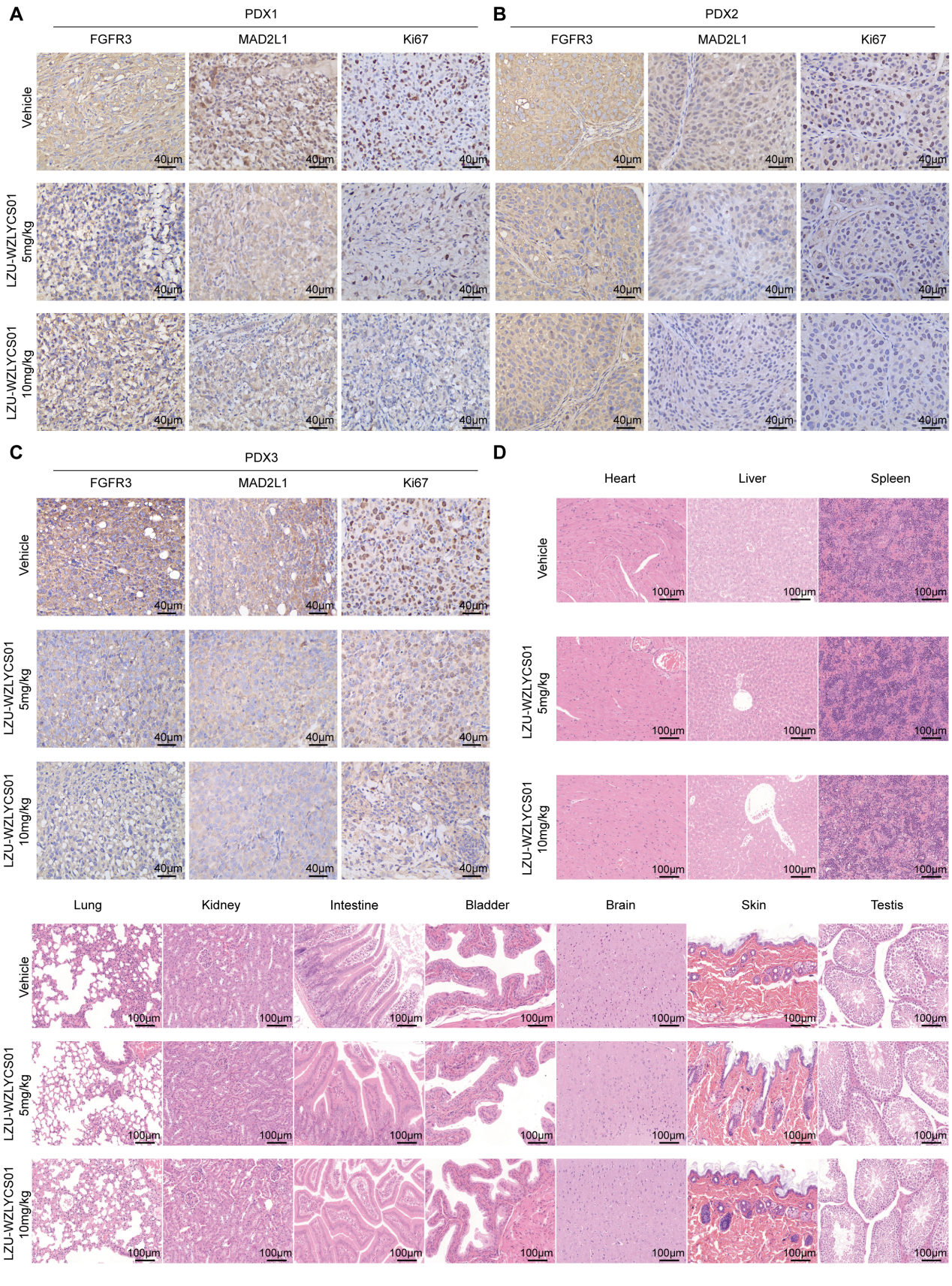


**Figure S10. IHC analysis of tumor tissues and histopathological evaluation of major organs following **LZU-**WZLY**CS01** treatment. A-C** Representative IHC staining of FGFR3, MAD2L1, and Ki-67 in PDX1 **(A)**, PDX2 **(B)**, and PDX3 tumors **(C)**. Scale bar = 40 μm. **D)** Representative H&E staining of major organs (heart, liver, spleen, lung, kidney, intestine, bladder, brain, skin, and testis). scale bar = 100 μm.

**Table S1.** **Hematological parameters in NCG mice following LZU-CS01 treatment.**

|  | Vehicle | LZU-CS01(5mg/kg) | LZU-CS01(10mg/kg) | Units | Reference range |
| --- | --- | --- | --- | --- | --- |
| WBC | 2.6 | 2.6 | 1.8 | 10**^**9/L | 0.8-6.8 |
| Lymph# | 0.9 | 1.3 | 0.8 | 10**^**9/L | 0.7-5.7 |
| Mon# | 0.1 | 0.1 | 0.0 | 10**^**9/L | 0.0-0.3 |
| Gran# | 1.6 | 1.2 | 1.0 | 10**^**9/L | 0.1-1.8 |
| Lymph% | 35.2 | 50.6 | 43 | % | 55.8-90.6 |
| Mon% | 4 | 2.5 | 3.3 | % | 1.8-6.0 |
| Gran% | 60.8 | 46.9 | 53.7 | % | 8.6-38.9 |
| RBC | 8.90 | 9.37 | 8.46 | 10**^12**/L | 6.36-9.42 |
| HGB | 154 | 160 | 146 | g/L | 110-143 |
| HCT | 46.4 | 48.3 | 43.2 | % | 34.6-44.6 |
| MCV | 52.2 | 51.6 | 51.1 | fL | 48.2-58.3 |
| MCH | 17.3 | 17 | 17.2 | pg | 15.8-19.0 |
| MCHC | 331 | 331 | 337 | g/L | 302-353 |
| RDW | 14.9 | 14.2 | 14.8 | % | 13.0-17.0 |
| PLT | 1778 | 1728 | 1599 | 10**^**9/L | 450-1590 |
| MPV | 4.7 | 5.0 | 4.7 | fL | 3.8-6.0 |
| PDW | 16.2 | 16.3 | 16.4 | NA | NA |

**Table S2. Blood biochemical analysis in NCG mice following LZU-CS01 treatment.**

|  | Vehicle | LZU-CS01(5mg/kg) | LZU-CS01(10mg/kg) | Units | Reference range |
| --- | --- | --- | --- | --- | --- |
| ALB | 31.30 | 33.60 | 32.30 | g/L | 25-48 |
| TP | 54.20 | 56.30 | 53.30 | g/L | 36-66 |
| GLO | 22.90 | 22.70 | 21.00 | g/L | 5-40 |
| A/G | 1.40 | 1.50 | 1.50 | NA | NA |
| BUN | 7.23 | 6.01 | 6.93 | mmol/L | 4.9-10.4 |
| CRE | 30 | 70 | 32 | μmol/L | 26-88 |
| BUN/CRE | 60 | 21 | 54 | NA | NA |
| ALT | 30 | 36 | 32 | U/L | 28-132 |
| TBIL | 2.24 | 8.58 | 3.42 | μmol/L | 1.7-15.4 |
| ALP | 66 | 64 | 81 | U/L | 62-209 |
| CK | 849 | 953 | 833 | U/L | 68-1070 |
